# Improving the production efficiency of high-titania slag in Ti extraction process: fluxing effect on formation of pseudobrookite

**DOI:** 10.1038/s41598-020-63532-4

**Published:** 2020-04-16

**Authors:** Dong Hyeon Kim, Jung Ho Heo, Hyun Sik Park, Jin Kyung Kim, Joo Hyun Park

**Affiliations:** 1 Department of Materials Engineering, Hanyang University, Ansan, 15588 Korea; 2 Research and Development Center, Dongkuk Steel, Pohang, 37873 Korea; 3 Research and Development Center, LS-Nikko Copper, Ulsan, 44997 Korea; 4 Resources Recovery Research Center, Korea Institute of Geoscience and Mineral Resources (KIGAM), Daejeon, 34121 Korea; 5 Department of Materials Science and Engineering, KTH Royal Institute of Technology, Stockholm, 100 44 Sweden

**Keywords:** Materials for energy and catalysis, Structural materials

## Abstract

We investigated the carbothermic reduction process of ilmenite ore at 1873 K with flux addition. Without flux, the pseudobrookite phase with a high melting temperature was precipitated during ilmenite smelting. This could be the main reason for decreased reduction of iron in ilmenite. To accelerate reduction of ilmenite, two factors were considered. One is increasing the reduction driving force during smelting. Activity of FeO is the major factor to control reduction in driving force. The other factor is delay in formation of the pseudobrookite phase, a high-melting point precipitation phase. In this system, MgO in ilmenite could be used to form pseudobrookite. To control these factors, in this study, flux agent (i.e., Na_2_O or SiO_2_) addition was considered. The thermochemical simulation program, FactSage^TM^7.0 was used to calculate the viscosity of slag and the activity of components as fluxing agents were added. High-temperature experiments using an induction furnace were also conducted to confirm the computational results. To determine the composition of final products, i.e., titanium slag, X-ray fluorescence analysis was executed. As a result of Fe and Ti behaviours in slag, SiO_2_ addition showed no significant difference from the slag without flux. However, Fe reduction in ilmenite, i.e. TiO_2_-enrichment, was more accelerated when Na_2_O was added. X-ray diffraction, scanning electron microscopic and transmission electron microscopic analyses results also showed that even 1 wt% Na_2_O addition significantly influenced the titanium slag production compared to no flux addition.

## Introduction

As titanium has good physical and chemical properties, e.g. excellent strength, corrosion resistance, fatigue resistance, fracture toughness, and high-temperature characteristics, its demand steadily increases^1–5^. The major raw material for titanium extraction is rutile (~95% TiO_2_), which quickly is being depleted. Therefore, ilmenite (FeTiO_3_, 30–65% TiO_2_) as a substitutional resource is in the spotlight. There are previous studies about the ilmenite smelting process in conjunction with pre-oxidation treatment, which improves the reactivity of ilmenite^6–13^. After the pre-oxidation treatment, the ilmenite ore is sent to direct leaching or high temperature reduction stages according to the consecutive processes^14–18^.

Our group originally investigated the effect of temperature on FeO reduction, i.e., TiO_2_-upgrading, from ilmenite ore from 1723 to 1923 K^13^. Unlike the expectation that the Ti-yield would be greater with higher temperatures, the slag composition in the actual experiment was not significantly changed. One of the reasons is precipitation of pseudobrookite (ps-BR), which is one of the most stable phases with M_3_O_5_ stoichiometry. In the position of M, the divalent cations (mainly Fe^2+^, minor Ti^2+^ and/or Mg^2+^) and the trivalent cations (primarily Ti^3+^) are in balance of valence. Thus, it is normally expressed as [Fe,Ti,Mg]_*x*_Ti_*y*_O_5_ (*x* + *y* = 3) formula. Because the melting point of ps-BR increases with decreasing Fe^2+^ content during reduction, the precipitation of ps-BR prohibited further reaction due to a sharp increase in slag viscosity at reduction temperature to 1923 K^13^.

Alternatively, the melting point of ps-BR increases by partial substitution of Mg^2+^ ion for Fe^2+^ ion during reduction, which is deleterious to TiO_2_-upgrading process. In the present study, we focused on the energy saving process by considering two factors to improve the efficiency of ilmenite reduction reaction. One is decreasing the chemical potential of MgO in slag to supress (or delay) the precipitation of ps-BR. The other is increasing the activity of FeO in slag as a driving force in the smelting reduction process. Furthermore, the higher the activity of FeO, the lower the viscosity of slag is expected, which improve the reaction kinetics.

To manage these factors, flux addition in ilmenite slag was investigated. There are previous studies for the effect of flux additives on the efficiency of TiO_2_-upgrading process^19–21^. Fan *et al*.^19^ investigated the addition of B_2_O_3_ during synthetic rutile production with molten TiO_2_-slag. The CaSiO_3_ and MgTi_2_O_5_ phases formed during slag melting were changed to Ca_2_B_2_O_5_ and MgB_2_O_6_, respectively, by addition of B_2_O_3_. These phases were leached out by HCl, leading to high-purity TiO_2_-slag. Song *et al*.^20^ studied the effect of Na_2_B_4_O_7_ on carbothermic reduction of ilmenite concentrate and confirmed that only 2 wt% of Na_2_B_4_O_7_ could improve the reduction rate by accelerating the metallization ratio of iron in the ilmenite concentrate. Also, Guo *et al*.^21^ found that a small amount of borax could enhance the reduction of Panzhihua ilmenite by affecting mineral phase transformation, microstructures, melting point, and Mg distribution. Alkali roasting of the ilmenite process, which is a pre-treatment of the hydrometallurgical process for obtaining high titanium yields, has been studied by several groups^22–25^. Sodium and potassium carbonates are more efficient for iron removal than lithium carbonate^23^. The effect of soda ash (sodium carbonate) ratio on ilmenite reduction was tested at a fixed roasting temperature by Lasheen^24^.

However, most of the above studies have been conducted at temperatures up to 1673 K, which is relatively lower than the ilmenite smelting temperature. Moreover, there have been no investigations of fluxing effect under ilmenite smelting conditions. Therefore, in the current study, we attempted to maximize the reduction of FeO from ilmenite by controlling the physicochemical factors affecting the reduction kinetics with fluxing method.

## Materials and Methods

### Materials preparation

The average particle size of ilmenite ore was less than 100 μm. The composition of raw ilmenite was investigated by X-ray fluorescence spectroscopy (XRF; ZSX Primus II, Rigaku, Japan), and X-ray diffraction (XRD; D/MAX-2500/PC, Rigaku, Japan) was used to confirm the phase. The fluxing additives such as SiO_2_ (one of strong acidic oxides) and Na_2_O (one of strong basic oxides) are regent grade chemicals of 99% purity^26^. Na_2_O was added in the form of Na_2_CO_3_, during which CO_2_ can be removed by thermal decomposition reaction, i.e., Na_2_CO_3_(s) = Na_2_O(l) + CO_2_(g). The amount of additive was designed from 1 to 9 wt%, and the ilmenite ore and flux powders were mixed homogeneously. The chemical composition of the ilmenite ore used in this study is listed in Table 1. It is clarified that it is composed mainly of ilmenite and hematite phases, while the fraction of rutile and iron-based ps-BR (Fe_2_TiO_5_) phases are insignificant through XRD analysis. The XRD pattern of raw ilmenite ore is given in Supplement 1.

**Table 1:** Chemical composition of ilmenite (wt%).

Ti	Fe	Si	Al	Mn	Mg	O
32.6	28.7	1.9	1.3	1.1	0.4	33.9

### Experimental procedure

The present experiments were carried out using a high-frequency induction furnace, a schematic of which is shown in our previous article and is provided in Supplement 2^13^. The details of furnace specification, chamber dimension, impurity removal method from gases, and details of experimental procedure are available elsewhere^13^. Electrolytic iron (100 g) was initially placed in a graphite crucible (outer diameter; 60 mm, inner diameter; 42 mm, height; 120 mm), used as both a refractory material and as a source of reductant. The details for the choice of graphite as a container in the present study are available elsewhere^13^.

The experimental temperature was 1873 K and was controlled within ±2 K using a B-type (Pt-30%Rh/Pt-6%Rh) thermocouple and a proportional integral differential controller. After the temperature stabilized, a mixture of ilmenite ore and flux (100 g) was quickly added onto the surface of the pre-melted iron bath and maintained for 1 h. Slag samples were collected at defined time intervals (5, 7, 10, 15, 20, 30 and 60 min) using a steel rod dipped into the slag layer. The samples were quenched rapidly by flushing with high-purity (99.999%) Ar gas (flow rate of 25 L/min). The quenched slag samples were crushed to powders using a steel mortar, followed by a sieving in size of under 100 μm for XRD and XRF analyses. The chemical composition of final titanium slags after 60 min experiments are listed in Table 2.

**Table 2:** Chemical composition of final slag after smelting reduction experiments (wt%).

Sample ID (Initial Flux)	TiO_2_	FeO	SiO_2_	Al_2_O_3_	MnO	MgO	Na_2_O
No flux	79.8	15.1	1.2	1.4	1.7	0.6	—
3%SiO_2_	78.4	12.4	3.2	1.9	2.2	1.8	—
6%SiO_2_	76.8	12.4	5.4	1.9	2.8	0.4	—
9%SiO_2_	75.4	10.2	9.9	1.7	2.2	0.4	—
1%Na_2_O	89.0	6.8	0.9	0.4	2.4	0.1	0.1
3%Na_2_O	88.7	3.0	1.7	1.6	1.6	0.9	2.2
6%Na_2_O	90.3	2.2	1.1	1.3	0.6	0.9	3.3
9%Na_2_O	91.4	2.4	1.6	2.3	0.3	0.9	0.8

### Characterization of samples

The chemical compositions of the slag samples were determined by XRF. The samples were then tested via XRD to confirm the phase of the slag, and field emission scanning electron microscopy with an energy dispersive X-ray spectroscope (FESEM-EDS; MIRA 3, TESCAN Ltd., Czech) was used to obtain the morphology of samples. Transmission electron microscopy (TEM; JEOL, JEM 2100 F) with a lattice resolution of 0.14 nm was used at 200 kV to investigate the crystallography of precipitated ps-BR phase in titanium slag. The specimen was thinned using focused ion beam (FIB; LYRA1, TESCAN Ltd., Czech) milling for TEM analysis.

## Results and Discussion

### Prediction of physicochemical change of ilmenite slag through thermodynamic simulation

As mentioned before, the type of fluxes was determined considering their effect on the activities of FeO and MgO in ilmenite slag. The carbothermic smelting reduction of FeO in ilmenite slag is represented by Eq. (1).

1 $$({\rm{FeO}})+{\rm{C}}({\rm{s}})={\rm{Fe}}({\rm{l}})+{\rm{CO}}({\rm{g}})$$

Hence, when FeO activity is high, the driving force of forward reaction is stronger, leading to TiO_2_-enrichment in the slag. Therefore, to increase the activity of FeO, the strong alkaline basic oxide such as Na_2_O was considered as catalytic fluxing agent.

Alternatively, the activity of MgO should be considered, because the incorporation of Mg^2+^ ion into the ps-BR phase, i.e., the formation of magnesium-based M_3_O_5_ phase (MgTi_2_O_5_) by Eq. (2)^27^, sharply increases the melting point and thus viscosity of slag.

2 $$({\rm{MgO}})+2({{\rm{TiO}}}_{2})={{\rm{MgTi}}}_{2}{{\rm{O}}}_{5}({\rm{s}})$$

It could be an obstacle for further reaction of TiO_2_-enrichment in ilmenite slag. Thus, the strong acidic oxide such as SiO_2_ was considered as potential fluxing agent to decrease the activity of MgO in the slag.

**Figure 1:**
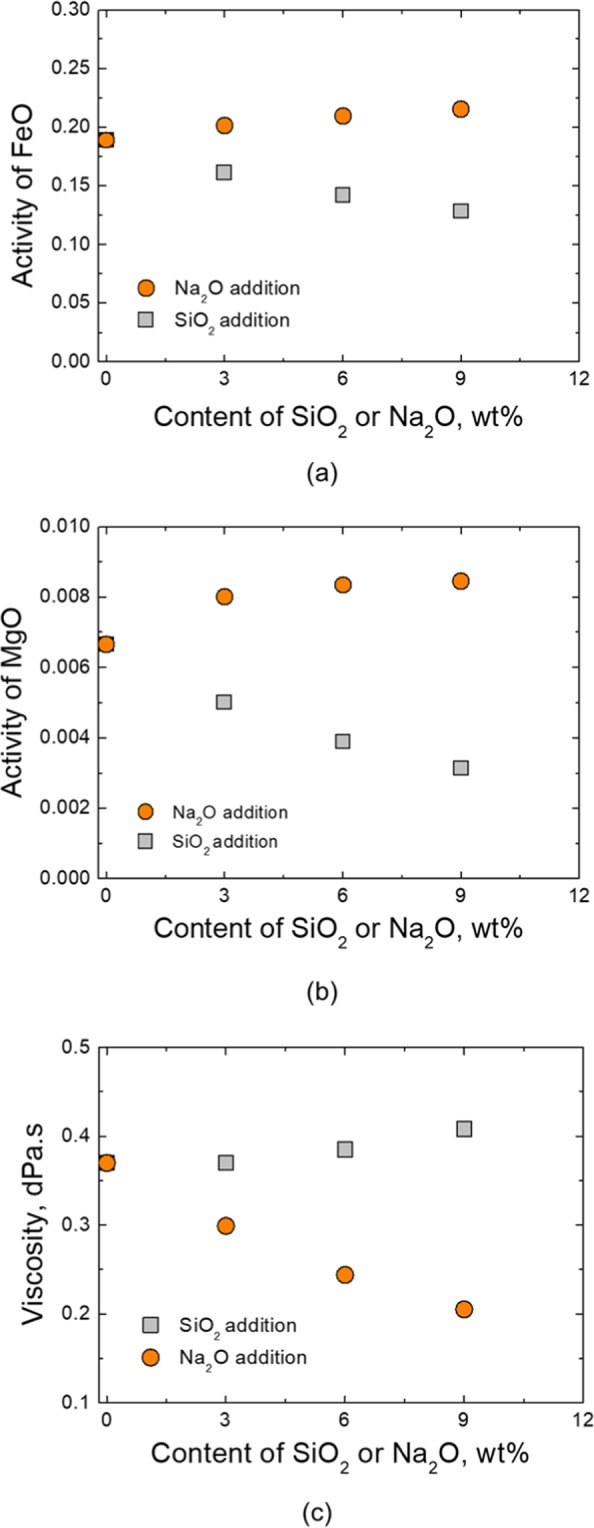
Change of (**a**) FeO activity, (**b**) MgO activity and (**c**) slag viscosity as a function of flux addition.

The change in activity of FeO and MgO as flux (Na_2_O or SiO_2_) addition was analysed using the thermochemical computing software, FactSage (version 7.0) as shown in Fig. 1. The activity of FeO linearly increases with increasing content of Na_2_O (Fig. 1a), while the similar tendency is predicted for MgO (Fig. 1b). The activity of FeO and MgO linearly decreases with increasing content of SiO_2_. Therefore, it is necessitated to experimentally confirm if each fluxing agent, Na_2_O or SiO_2_ would be effective or not in view of TiO_2_-enrichment efficiency.

Slag viscosity with flux was also simulated. Viscosity is useful information in the smelting process because slag with high fluidity is easier to promote the reaction kinetics. Pistorius *et al*.^8,9^ also mentioned that iron oxide is more easily reduced in the liquid phase during ilmenite smelting. Simulated data of viscosity using FactSage software is shown in Fig. 1c. The result shows viscosity has a slightly increasing tendency with SiO_2_ addition. Meanwhile, the viscosity is lowered rapidly with Na_2_O addition.

Even though the viscosity of titanium slag was investigated by several authors, it is difficult to understand the structure-viscosity relationship because the structure of titanate is significantly different from that of silicate melts. Recently, Hu *et al*.^28^ directly measured the viscosity of titanium slag using rotating viscometer and found that the viscosity decreases with increasing FeO, while it increases with increasing TiO_2_ content^28,29^. They also calculated the coordination number (CN) of titanium using molecular dynamic (MD) simulations, by which the CN of Ti in the TiO_2_-rich melts would be 6, i.e., the [TiO_6_]-octahedron is main backbone structure of the melts^28,29^. The MD simulation and viscosity measurements were also performed by Kim and Park for the FeO-TiO_2_ (-Na_2_O) systems^30^. They obtained the similar results with Hu *et al*.^28^ and Huang *et al*.^29^ for the FeO-TiO_2_ system and additionally found that Na_2_O decreases the viscosity of the melts, resulting from a decrease in CN of titanium by Na_2_O addition. A decrease in CN of titanium means the network breaking event by Na_2_O, resulting in a decrease in viscosity^30^.

Although there is no quantitative experimental results for the effect of SiO_2_ on the titanium slags, it is easy to expect that SiO_2_ could increase the viscosity of the titanium slag because SiO_2_ has strong covalent Si-O bonds and tends to form a polymerized [SiO_4_]-tetrahedron structure^31–35^. This could be the reason for a decrease in slag fluidity. From the viscosity point of view, we concluded that the addition of alkaline oxide can help to reduce FeO in ilmenite. Based on these calculated results, experiments on fluxing effect were executed.

### Change of Fe and Ti contents in ilmenite slag by fluxing method

**Figure 2:**
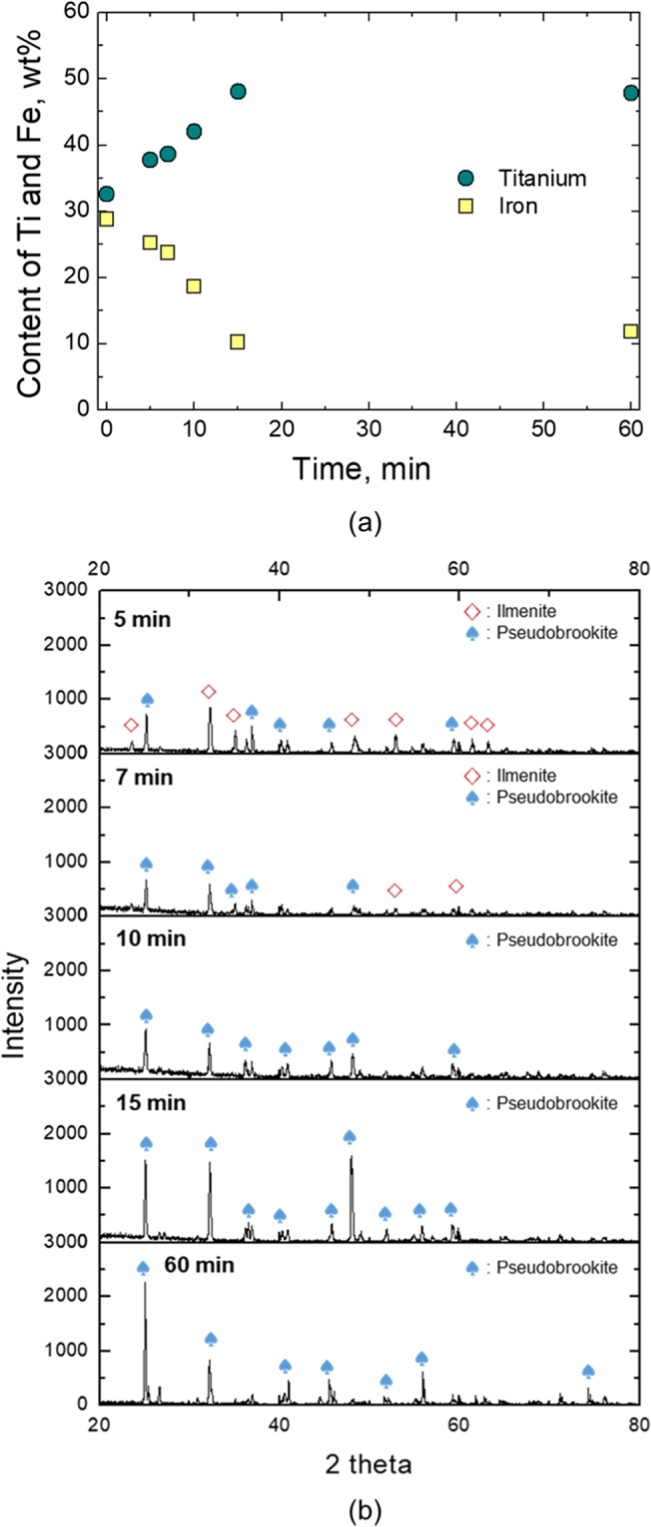
(**a**) Change of Ti and Fe content in slag without flux addition and (**b**) XRD pattern of slag samples with time during reduction process at 1873 K (Refinement of XRD peak identification is given in Supplements 3).

Before the flux addition experiments, the experiment without additive was performed, and slag was sampled at defined time intervals. The contents of Fe and Ti were analysed by XRF, and the phase of sampled slag was determined by XRD patterns. The refinement of peak identification was performed using an analysis software with a mineral database (MDI JADE 9.0) and the relevant data are provided in Supplement 3. The results are shown in Fig. 2. The Fe content rapidly decreased for the first 15 min, after which, however, there was no significant difference in Fe and Ti contents to completion of the experiment (Fig. 2a). XRD analysis showed coexistence of ilmenite and ps-BR until 7 min. After that, only ps-BR phase was observed (Fig. 2b).

**Figure 3:**
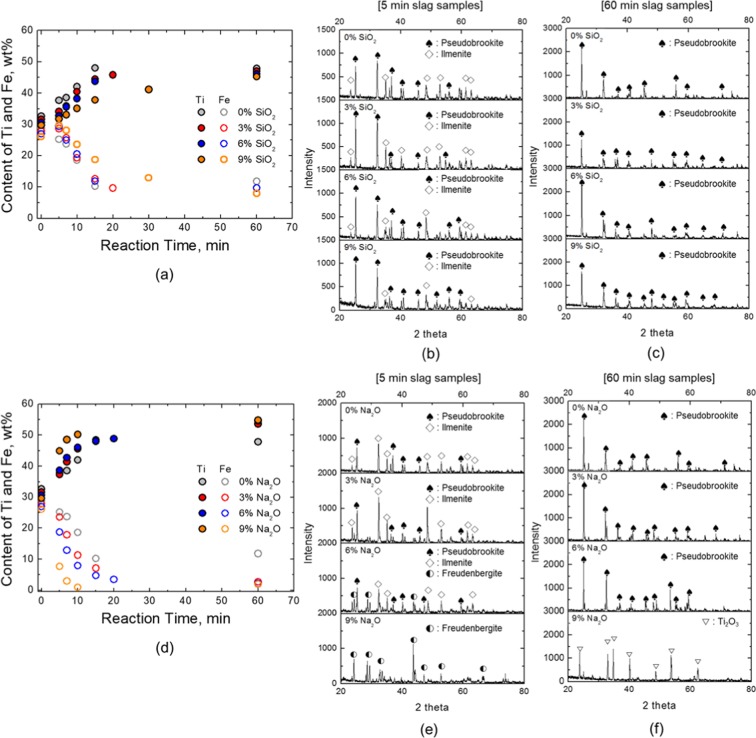
(**a**) Changes in Ti and Fe content in the slag and XRD pattern of the slag samples at (**b**) 5 min and (**c**) 60 min during SiO_2_ fluxing experiments, and (**d**) Changes in Ti and Fe content in the slag and XRD pattern of the slag samples at (**e**) 5 min and (**f**) 60 min during Na_2_O fluxing experiments (Refinement of XRD peak identification is given in Supplements 3).

Next, carbothermic ilmenite smelting with flux addition was executed. The experiments were conducted at 3, 6 or 9 wt% flux, and slag was sampled at defined time intervals. The composition of slag samples analysed by XRF is shown as a function of reaction time in Fig. 3. For SiO_2_ fluxing (Fig. 3a), Fe (or Ti) in ilmenite was rapidly reduced (or enriched) until 15 min and then was nearly constant under 0 to 6 wt% SiO_2_ conditions. In comparison, Fe (or Ti) was slowly and continuously reduced (or enriched) during the reaction with 9 wt% SiO_2_. After 60 min, the contents of Fe and Ti in titanium slag were not significantly different depending on additive presence.

However, the effect of Na_2_O fluxing on Fe reduction (or Ti enrichment) were quite different from that of SiO_2_ fluxing. Reduction occurred rapidly in the early stage of each experiment. But differences were noted according to amount of flux (Fig. 3d). As Na_2_O was added, much larger amounts of Fe were reduced, and the reduction rate increased rapidly. In particular, Fe (or Ti) content in titanium slag at 60 min was remarkably lower (or higher) than that with no flux addition. In addition, Fe and Ti contents at 3 wt% Na_2_O addition were not significantly different from those at 6 and 9 wt% addition.

To determine which phase was generated and to obtain slag morphology, XRD and SEM-EDS analyses were performed. The XRD patterns at 5 min and 60 min according to SiO_2_ and Na_2_O fluxing experiments are shown in Fig. 3b,c,e,f, respectively. The XRD analysis with SiO_2_ addition confirmed no remarkable difference from the XRD pattern without SiO_2_, and 5 min slag samples showed coexistence of ilmenite and ps-BR phases (Fig. 3b). Similarly, the titanium slag samples (60 min) showed a final composition of ps-BR regardless of the amount of SiO_2_ additive (Fig. 3c).

Conversely, the XRD pattern of the slag containing Na_2_O showed a different aspect from that of SiO_2_ fluxed slags. In the 5 min treated slags (Fig. 3e), the 3 wt% Na_2_O addition experiment showed a comparable pattern to that of the no addition experiment. However, when the amount of Na_2_O became larger than 3 wt%, Na-Ti-Fe contained mineral phase called freudenbergite (Na_2_Fe_2_Ti_6_O_16_) was formed and only freudenbergite existed at 9 wt% Na_2_O fluxed slag. For the titanium slag with 9 wt% Na_2_O (60 min), Ti_2_O_3_ was observed instead of the previously observed ps-BR phase (Fig. 3f). Formation of Ti_2_O_3_ is due to an excessive reduction of TiO_2_ to Ti_2_O_3_ as reaction time is extended^6,8,36^. The addition of Na_2_O promotes the reduction of ilmenite, resulting in an enhancement of the following reduction reaction:

3 $$2({{\rm{TiO}}}_{2})+{\rm{C}}({\rm{s}})=({{\rm{Ti}}}_{2}{{\rm{O}}}_{3})+{\rm{CO}}({\rm{g}})$$

Attempts to produce higher TiO_2_ slag resulted in the formation of Ti_2_O_3_, which is less soluble in sulphuric acid; hence, such slags are not suitable for sulphate process in pigment high-titania production^36,37^.

**Figure 4:**
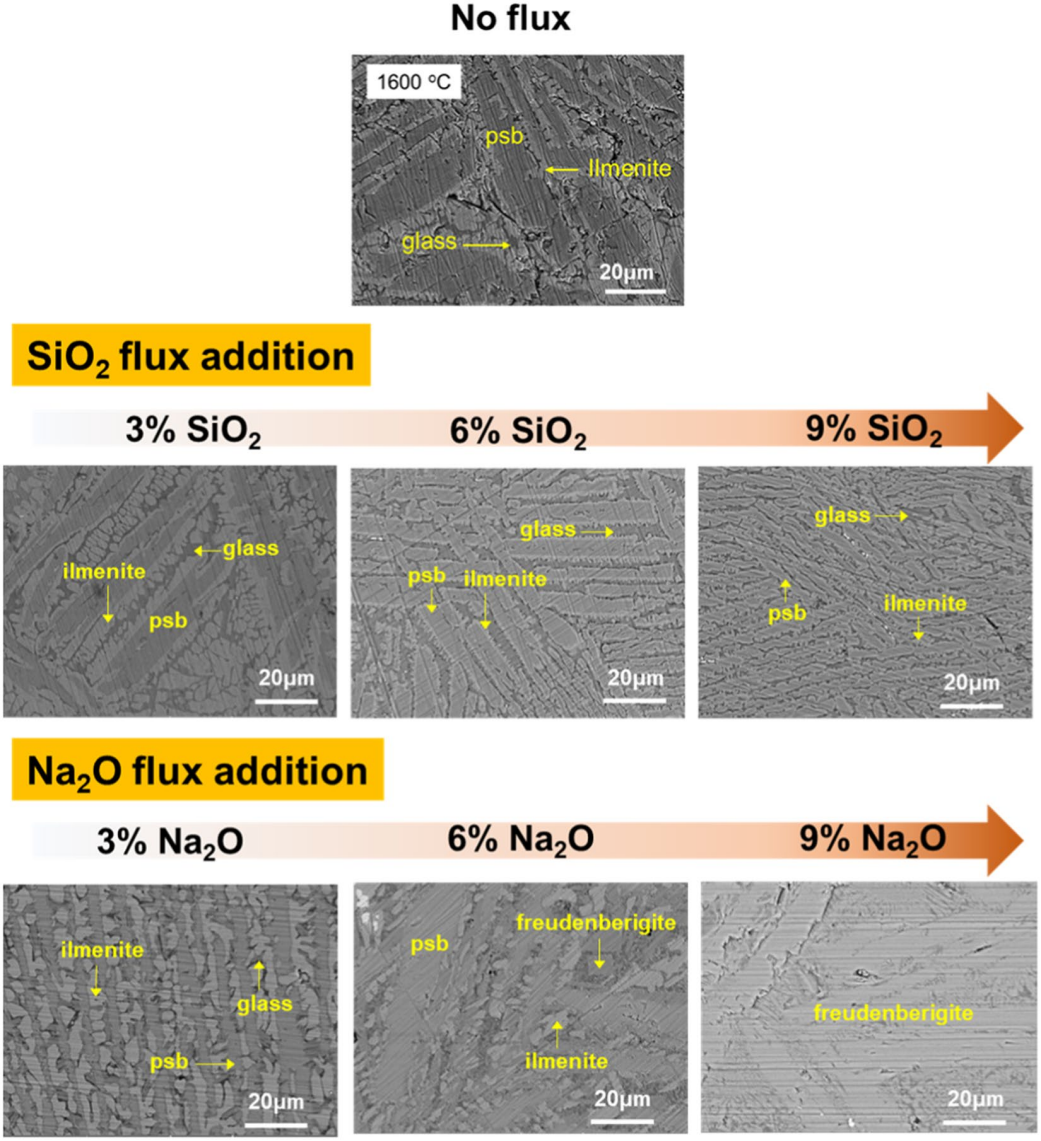
Back scattered electron images of quenched slags at 5 min for without flux, SiO_2_ and Na_2_O fluxing experiments (EDS spectra and element mapping results of each phase are given in Supplement 5 and Supplement 6, respectively).

The SEM images of sampled slag showed similar results as shown in Fig. 4. All corresponding EDS spectra and element mapping analysis results are provided in Supplement 4 and Supplement 5, respectively. In the SEM images for the SiO_2_ fluxed slags, the larger was the SiO_2_ content, the smaller was the size of the ps-BR crystallites. However, in the Na_2_O fluxed slags, the larger was the Na_2_O content to 6 wt%, the larger was the size of the ps-BR crystallites. Kirkpatrick investigated general crystal growth from melt and found that growth rate and viscosity were inversely related^38^. Growth rate slowed due to increased viscosity as SiO_2_ was added, resulting in dense crystallites.

**Figure 5:**
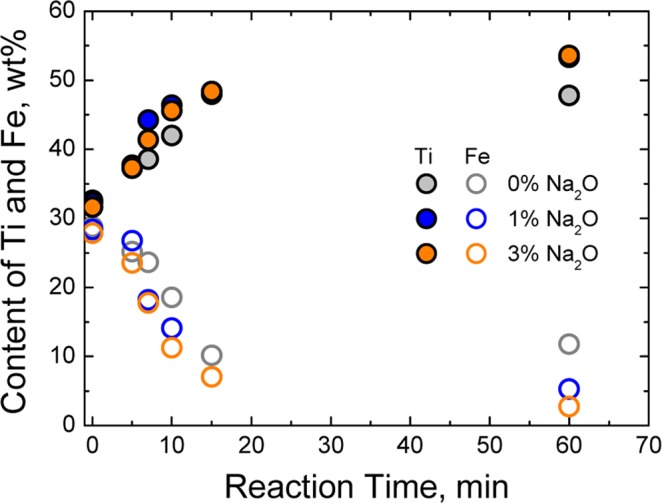
Variation of titanium and iron content in slags for Na_2_O fluxing experiments as a function of reaction time at 1873 K.

Based on the above experimental and analysis results, a Na_2_O 1 wt% addition experiment was carried out to determine if addition of at most 1 wt% Na_2_O fluxing could affect Fe reduction, i.e., TiO_2_-upgrading in the slag. The results are shown in Fig. 5, where the 1 wt% fluxing result is close to that of 3 wt% fluxing tendency during entire experiments. The 60 min slag treatment was noteworthy because, even though just 1 wt% Na_2_O was added, the content of Fe (or Ti) significantly decreased (or increased), compared to the slag composition when flux was not applied. Based on the present experimental results, even a very small amount (~ 1 wt%) of Na_2_O flux is effective to produce the titanium slag from ilmenite ore.

### Influence of fluxing agents on reaction kinetics

**Figure 6:**
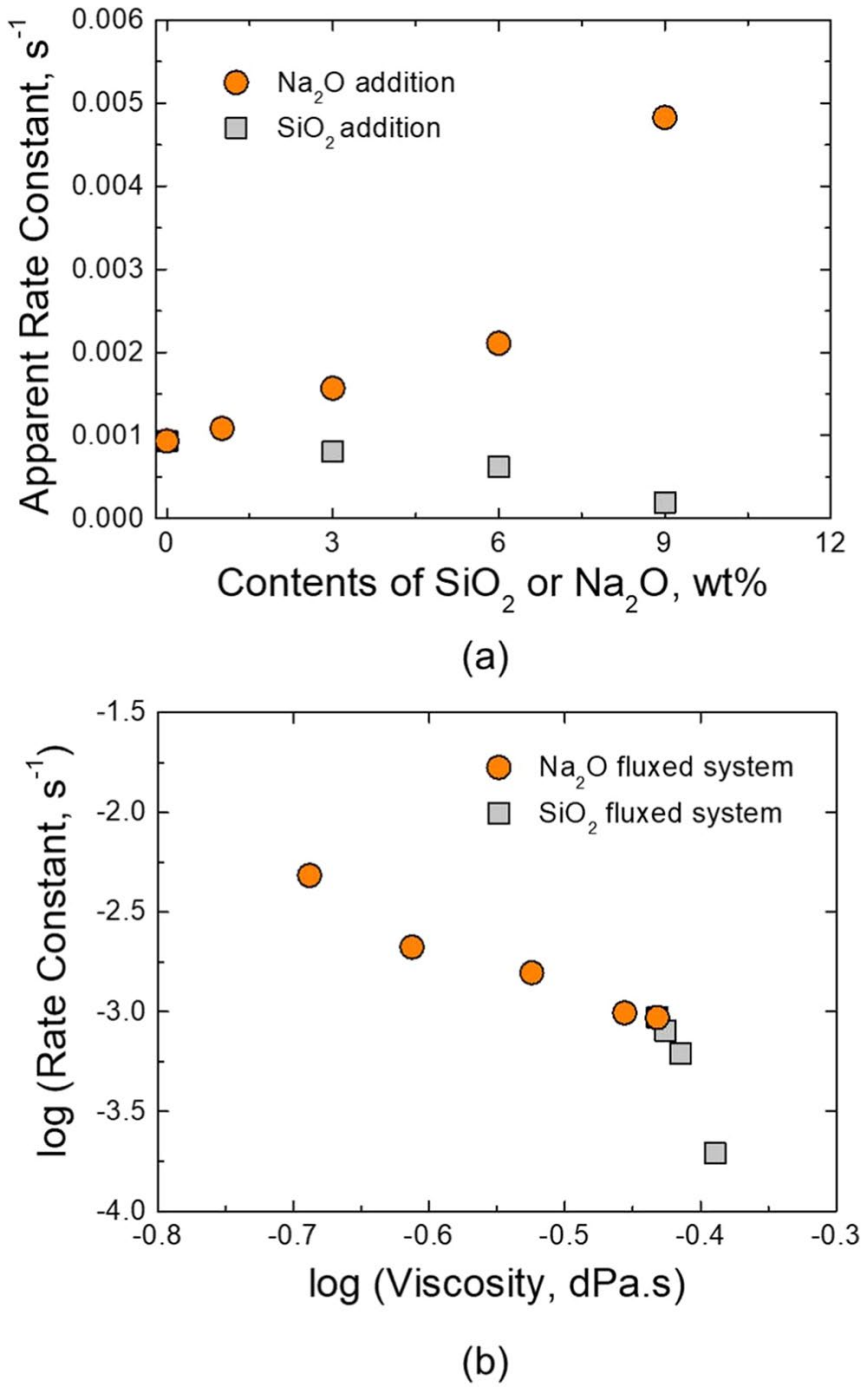
Apparent rate constant for iron reduction during ilmenite smelting process at 1873 K.

The reduction reaction of iron oxide in ilmenite by solid carbon originating from the graphite crucible is shown in Eq. (1). The effect of flux on the reduction kinetics was studied by the changes in Fe content in sampled slags, and the XRF results of each sampled slag were used for kinetic analysis. Due to the intense reduction reactions at slag-crucible interface as well as at slag-metal interface, it was difficult to determine the exact reaction interfacial area and volume. For this reason, the apparent rate constant ${k}_{app}({s}^{-1})$ was used for kinetic analysis of the reaction. The kinetic model used in this study, which is based on the apparent rate equation, is expressed as follows^39,40^:

4 $$-\frac{d( \% Fe)}{dt}={k}_{app}\cdot [{( \% Fe)}^{b}-{( \% Fe)}^{i}]$$

where ${( \% Fe)}^{b}$, ${( \% Fe)}^{i}$ are the Fe content in the bulk slag and at the interface, respectively. The apparent rate constant can be obtained from the change in Fe content in slag by integrating Eq. (4) with time by assuming that the content of Fe at the interface, *i.e*., ${( \% Fe)}^{i}$, is zero because of infinite supply of carbon. This assumption leads to Eq. (5).

5 $$-\,\mathrm{ln}\,\frac{{( \% Fe)}^{t}}{{( \% Fe)}^{o}}={k}_{app}\cdot t$$

where ${( \% Fe)}^{t}$ and ${( \% Fe)}^{o}$ are the Fe content at time $t$ and initial Fe content, respectively. Based on this 1^st^ order kinetic model, which was proposed by many researchers in smelting reduction of FeO by solid carbon in the slag under conditions of electric arc furnace and basic oxygen furnace processes^40–43^, the apparent rate constant for the reduction of FeO in the present slag system with each flux were calculated and plotted in Fig. 6a. The apparent rate constant rapidly increased as Na_2_O was added, while it slightly decreased with SiO_2_ addition.

It is noticeable that this result is in good correspondence to the viscosity change of the slag with each flux system as shown in Fig. 6b. Consequently, viscosity and apparent rate constant are closely related, and this result indicates that the Fe reduction and thus Ti-enrichment process are controlled by slag phase mass transfer^13,40^. In the previous study by the present authors^13^, the activation energy of the reaction was 144 kJ/mol, from which the slag phase mass transfer was deduced as the rate controlling step.

The present findings are qualitatively in good agreement with the previous results for the reaction mechanism of FeO in smelting reduction processes^40–43^. The reduction of FeO in the calcium silicate base slags are known to be controlled by the diffusional mass transfer of FeO (Fe^2+^ and O^2−^) in the slag phase at low or moderate FeO concentration range, while the reaction is generally controlled by the gas-carbon chemical reactions including Boudouard reaction at relatively high FeO concentration range. However, there are still room for further investigations to reveal the quantitative effect of elementary reaction step on the reduction mechanism of FeO in the slag under ilmenite smelting conditions.

### Identification of pseudobrookite (ps-BR) phase via FIB-TEM analysis

The structure of final titanium slag was generally identified as ps-BR phase, but the addition of a small amount of Na_2_O resulted in higher Ti-yield in the products. To investigate how Na_2_O flux affects the Ti content in ps-BR phase, TEM analysis was conducted. The titanium slag samples (60 min) of untreated and 3 wt% Na_2_O fluxed were prepared using the focused ion beam (FIB) system.

**Figure 7:**
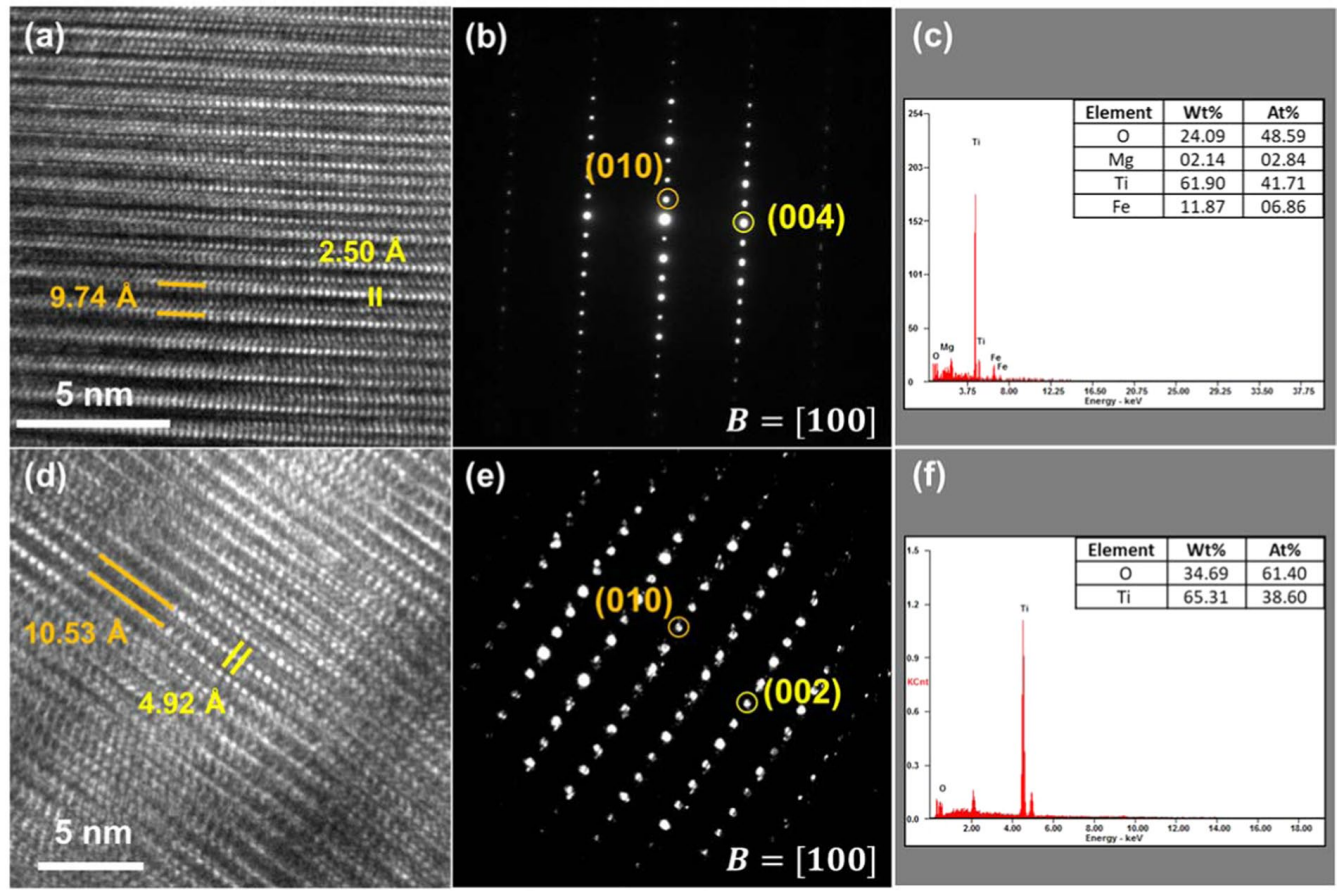
(**a**) High resolution (HR) image, (**b**) diffraction pattern (DP) and (**c**) EDS spectrum of ps-BR phase in untreated slag sample, and (**d**) HR image, (**e**) DP of and (**f**) EDS spectrum of ps-BR phase in Na_2_O-fluxed slag sample.

The high resolution TEM (HR-TEM) images, diffraction patterns and EDS spectra of the two investigated titanium slag samples are shown in Fig. 7. As shown in Fig. 7a (for untreated system) and Fig. 7d (for 3 wt% Na_2_O fluxed system), the HR-TEM images showed periodic lattice fringes. The TEM observation of the untreated titanium slag was conducted at the zone axis of $[100]$. The measured interplanar spacing of (010) plane and (004) plane is 9.740 Å and 2.499 Å, respectively (Fig. 7a). The TEM observation of the Na_2_O-fluxed titanium slag was also conducted at the zone axis of $[100]$. The interplanar spacing of (001) plane and (020) plane is 10.532 Å and 4.924 Å, respectively (Fig. 7d).

Both the HR-TEM images and corresponding diffraction patterns indicate that the structures of the investigated titanium slags correspond to an orthorhombic structure^44–47^. The Ti_3_O_5_, the ps-BR phase, was confirmed to have an orthorhombic structure (space group Cmcm, PDF# 01-089-4733), and the FeTi_2_O_5_ phase (space group Cmcm, PDF #01-076-2372) also had same crystal structure. The measured (010) interplanar spacing of the Na_2_O-fluxed titanium slag sample, 10.53 Å, is much higher than that of the untreated sample, 9.740 Å. The ideal (010) interplanar spacing of Ti_3_O_5_ and FeTi_2_O_5_ is 9.846 Å and 9.812 Å, respectively. Although the absolute values of the lattice parameter of each sample could not be precisely determined from HRTEM, we suggest that the structure of Na_2_O-fluxed titanium slag sample is closer to the structure of Ti_3_O_5_ since the ideal (010) interplanar spacing of Ti_3_O_5_ is larger than that of FeTi_2_O_5_.

The above findings are qualitatively in good agreements with the EDS analysis results for each sample as shown in Fig. 7c,f for untreated and Na_2_O-fluxed titanium slag samples, respectively. The magnesium and iron are remained in the former (Fig. 7c), while none of these impurities were detected in the latter (Fig. 7f). Thus, the analysis shows that the addition of a small amount of Na_2_O flux promotes reduction of Fe constituting the ps-BR phase, resulting in a high concentration of Ti in titanium slag and similar crystal structure and chemical composition with Ti_3_O_5_.

## Conclusions

In the present study, novel catalytic fluxing method were employed to accelerate the carbothermic smelting reduction of ilmenite ore at 1873 K through thermodynamic simulation and high temperature experiments. Two factors were considered, affecting the reduction of ilmenite: activities of FeO and MgO. To manage these factors, SiO_2_ and Na_2_O were added as catalytic fluxing agents. In the SiO_2_-fluxing experiments, reduction of FeO in ilmenite at the initial stage was similar regardless of fluxing amount (up to 6 wt%). There was no significant difference on the final composition of titanium slag with flux addition. However, in the Na_2_O-fluxing experiments, the reduction reaction was accelerated as the amount of Na_2_O flux increased. Hence, the final composition of titanium slag showed that FeO was highly reduced in the Na_2_O-fluxing experiments. As Na_2_O was supplied, the apparent rate constant showed a close relationship with viscosity. This means that the mass transfer of species was promoted by decreasing slag viscosity. Finally, TEM analysis shows that the addition of a small amount of Na_2_O flux promotes reduction of Fe constituting the ps-BR phase, resulting in a high concentration of Ti in titanium slag and similar crystal structure and chemical composition with Ti_3_O_5_.

## Supplementary information

Supplements Information.

## Data Availability

The datasets generated during the current study are available from the corresponding author on reasonable request.
