# Supplementary material for: Improving the production efficiency of high-titania slag in Ti extraction process: fluxing effect on formation of pseudobrookite
Source: Sci Rep. 2020 Apr 16;10:6530. doi: 10.1038/s41598-020-63532-4 (PMC7162870; doi:10.1038/s41598-020-63532-4)
Supplement: Supplementary file 1 — Supplements Information. [file 41598_2020_63532_MOESM1_ESM.pdf]

Improving the production efficiency of high-titania slag in Ti extraction process: fluxing effect on formation of pseudobrookite

Dong Hyeon Kim[1,2], Jung Ho Heo[1,3], Hyun Sik Park[4], Jin Kyung Kim[1] & Joo Hyun Park[1,5,*]

[1]Department of Materials Engineering, Hanyang University, Ansan 15588, Korea
[2]Research and Development Center, Dongkuk Steel, Pohang 37873, Korea
[3]Research and Development Center, LS-Nikko Copper, Ulsan 44997, Korea
[4]Resources Recovery Research Center, Korea Institute of Geoscience and Mineral Resources (KIGAM), Daejeon 34121, Korea
[5]Department of Materials Science and Engineering, KTH Royal Institute of Technology, Stockholm 11428, Sweden

*Correspondence and requests for materials should be addressed to J.H.P. (email: basicity@hanyang.ac.kr)

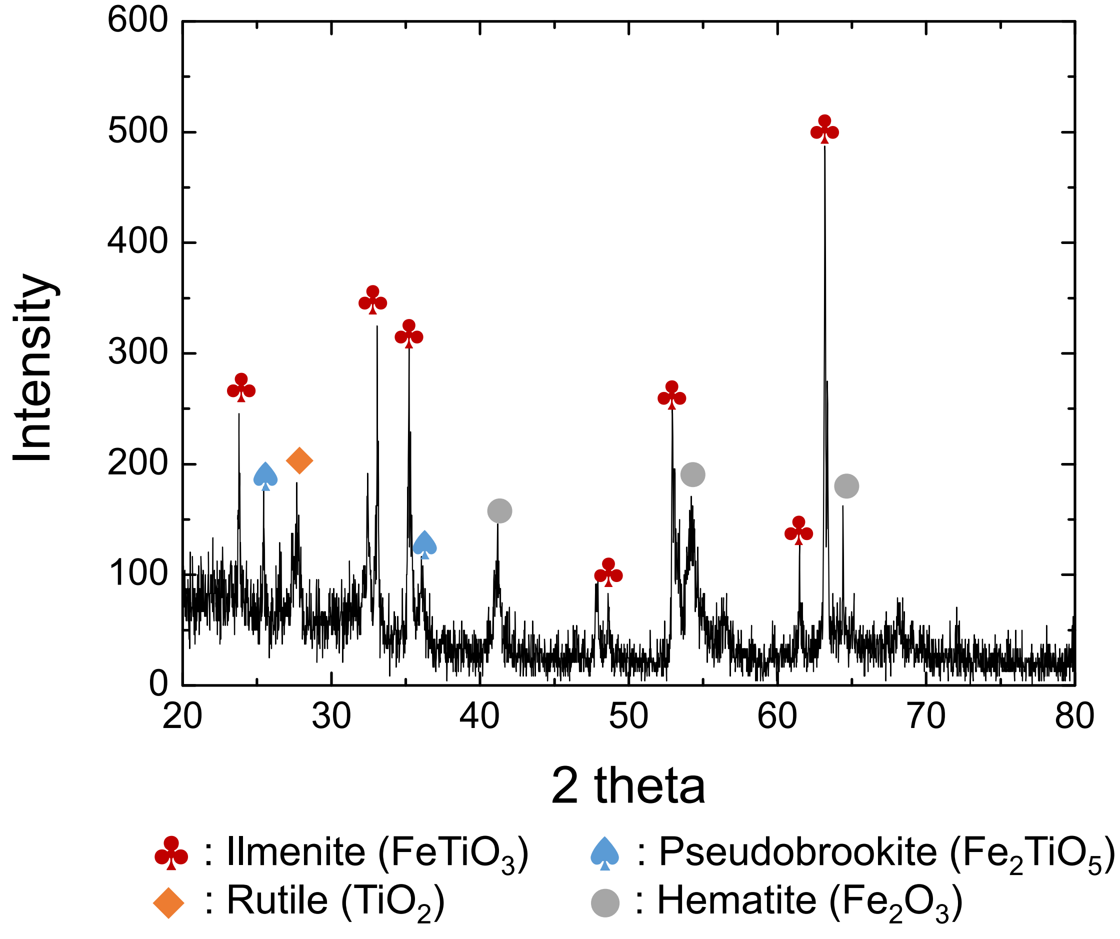

Supplementary 1. XRD pattern of raw ilmenite.

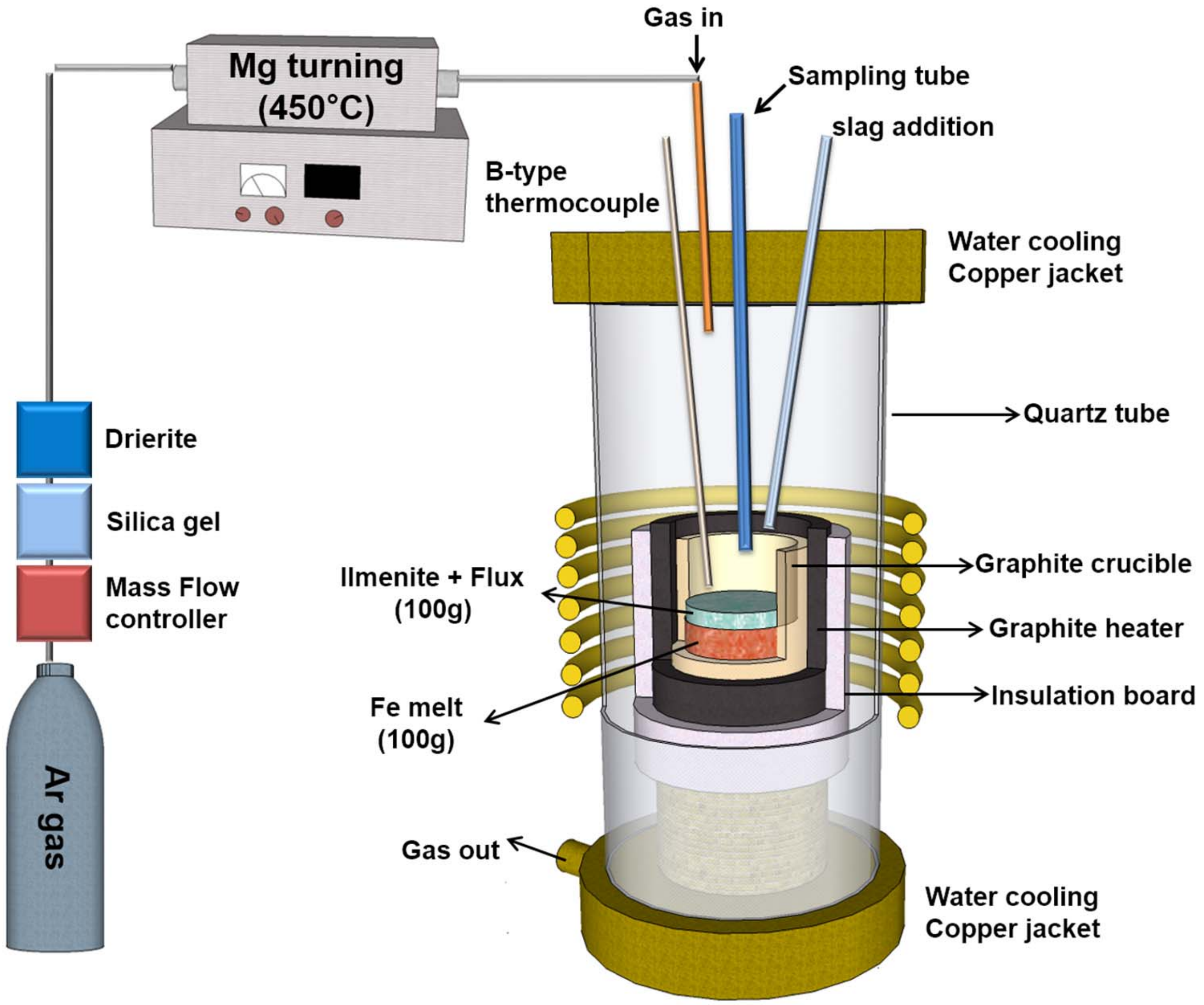

Supplementary 2. Schematics of experimental apparatus.[13]

No flux – 5min

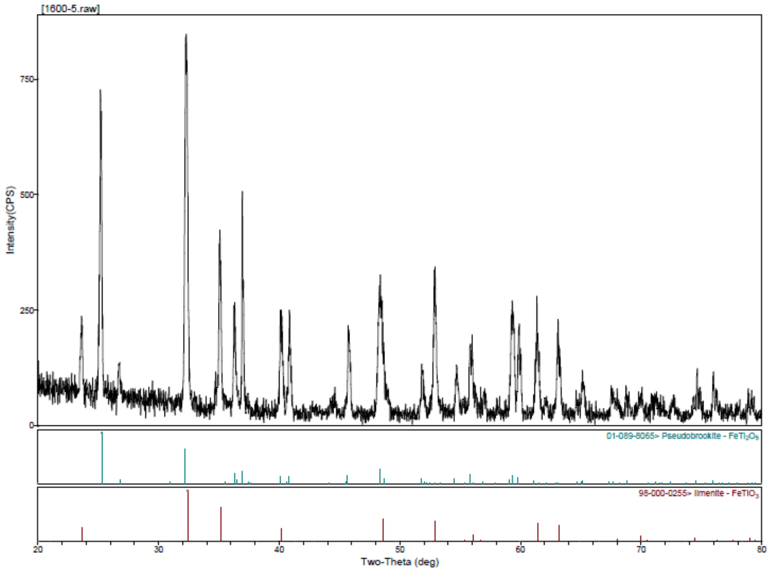

No flux – 7min

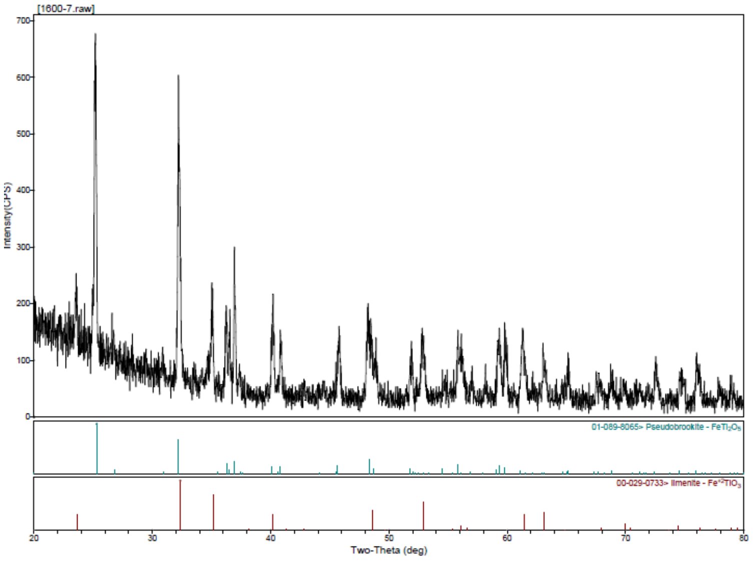

No flux – 10min

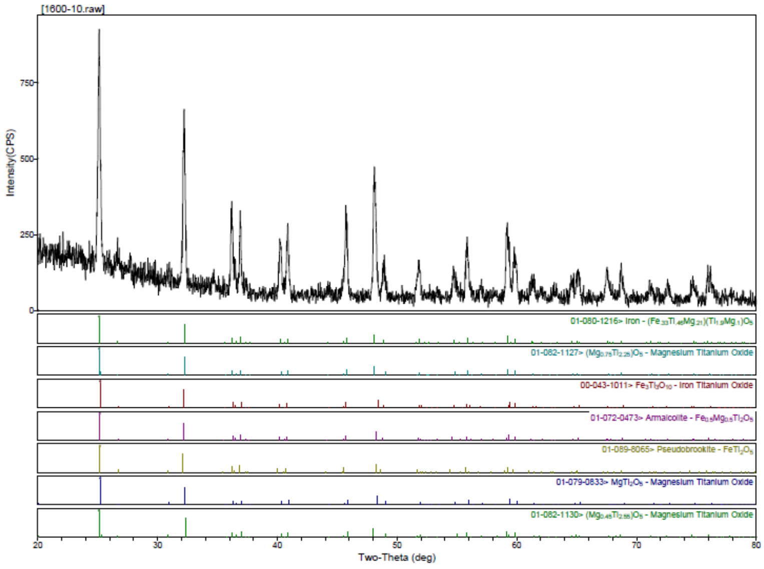

No flux – 15min

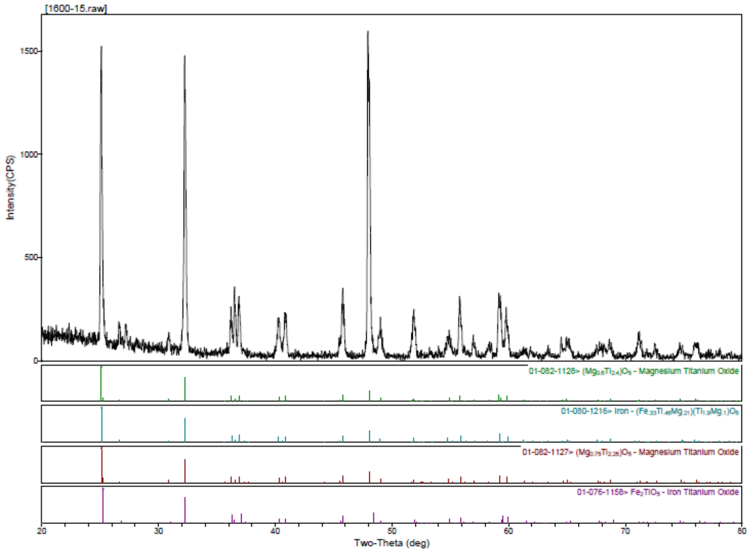

No flux – 60min

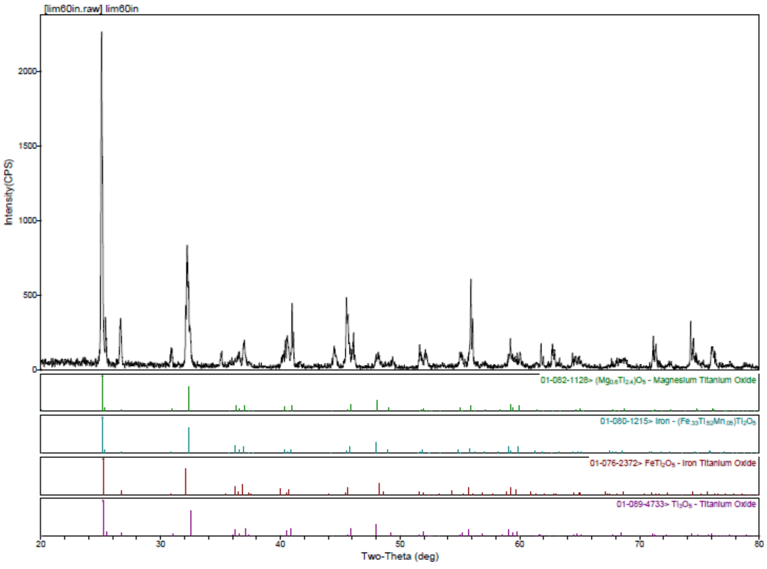

Supplementary 3. The refinement of each mineral phase in XRD pattern of slag samples shown in Fig 2b using JADE program.

3SiO2– 5min

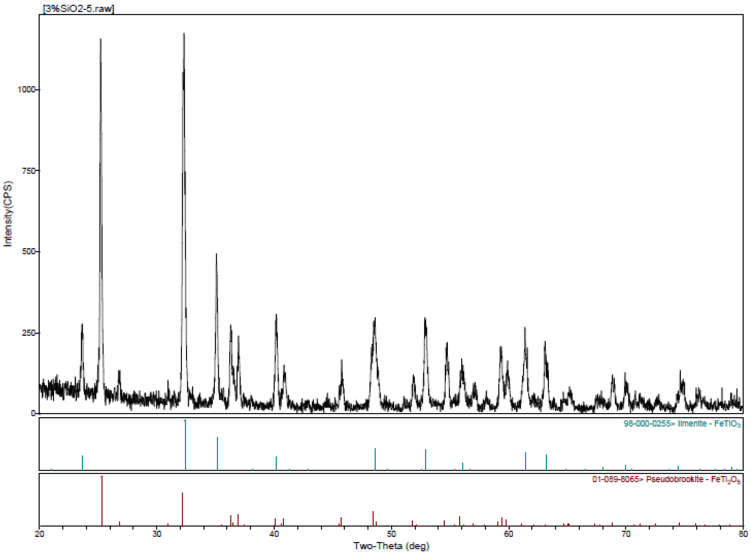

3SiO2– 60min

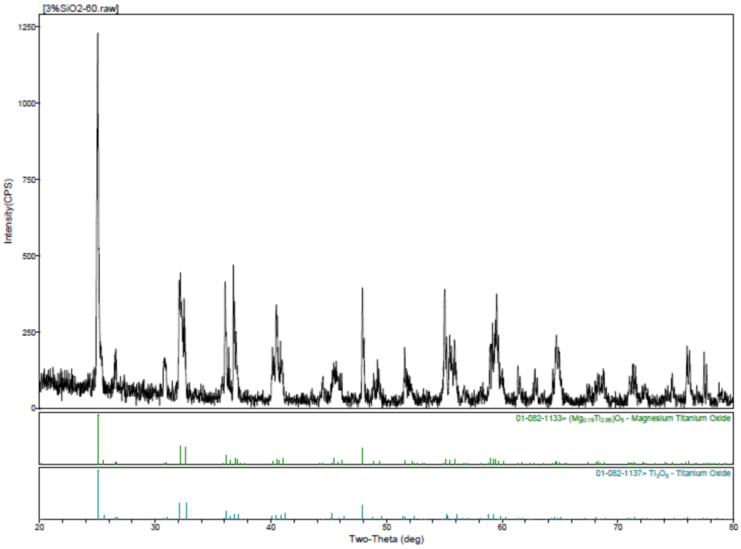

6SiO2 – 5min

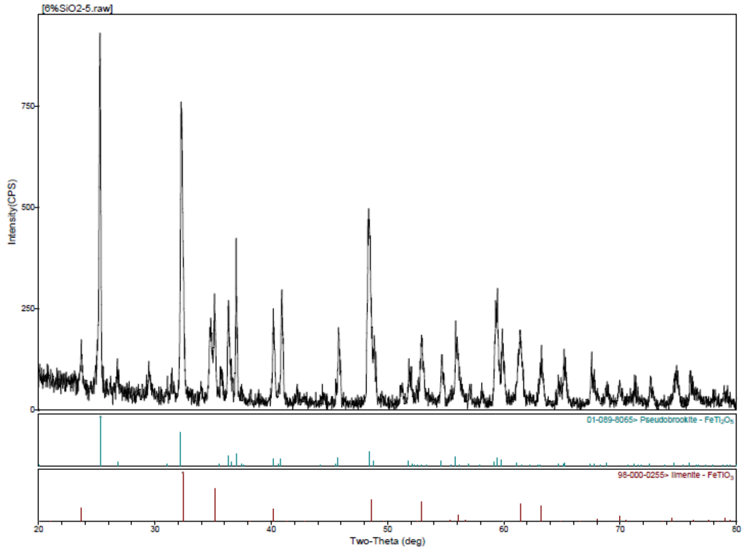

6SiO2 – 60min

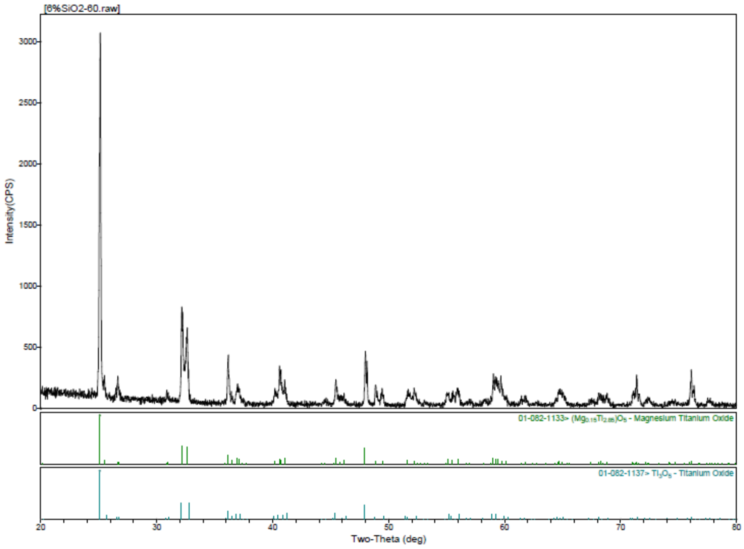

9SiO2 – 5min

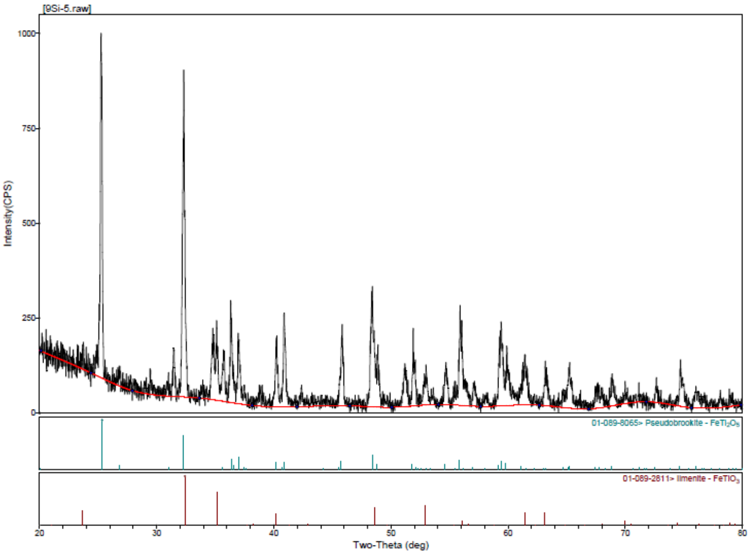

9SiO2 – 60min

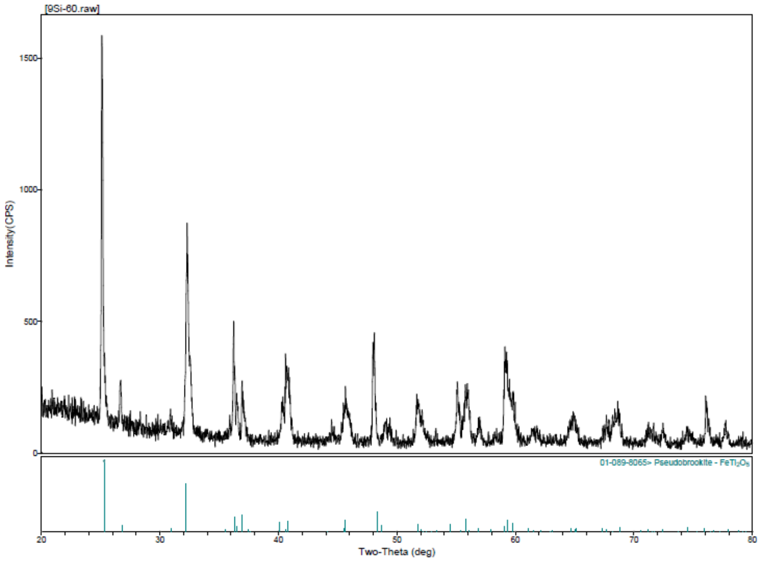

Supplementary 3. The refinement of each mineral phase in XRD pattern of slag samples shown in Fig 3b and 3c using JADE program.

3Na2O – 5min

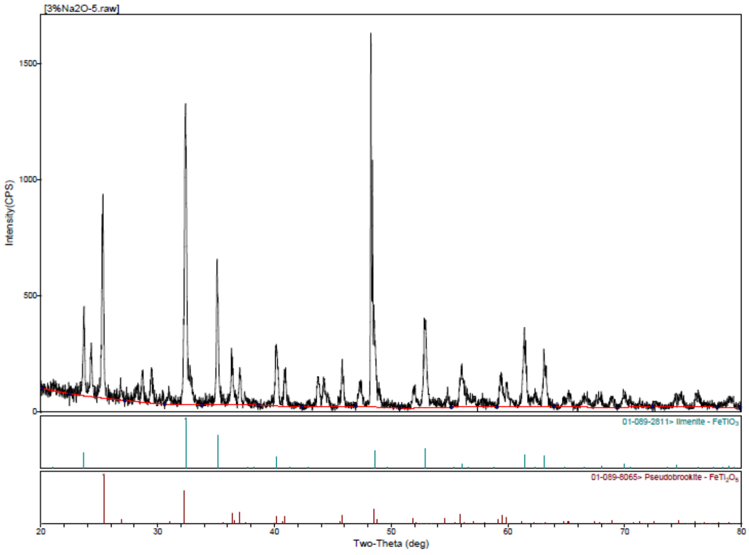

3Na2O – 60min

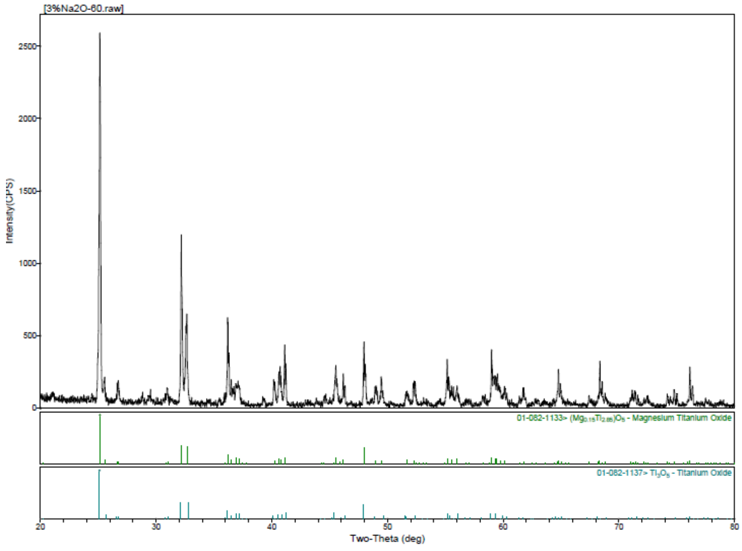

6Na2O - 5min

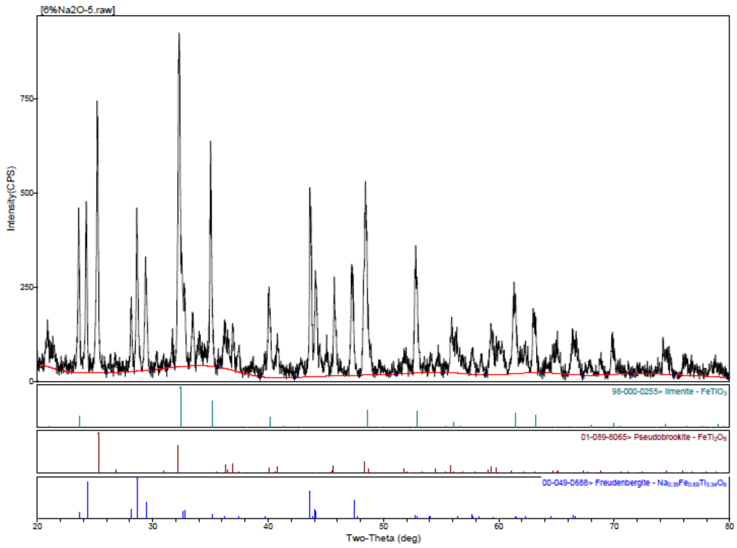

6Na2O - 60min

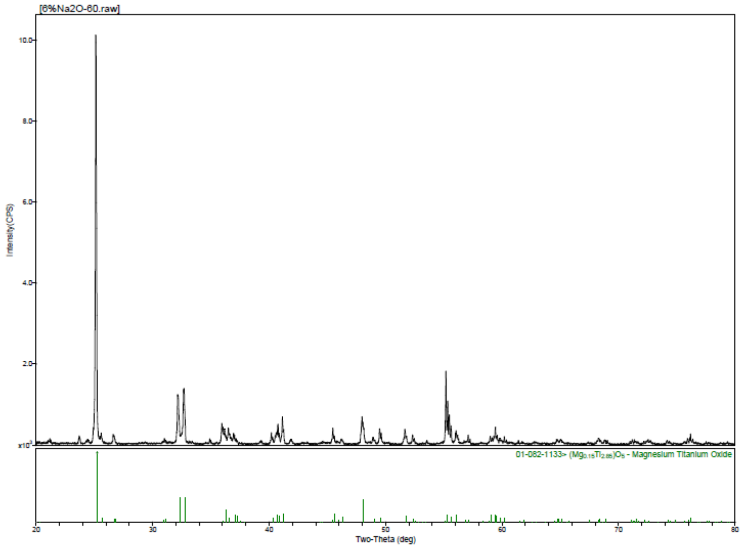

9Na2O – 5min

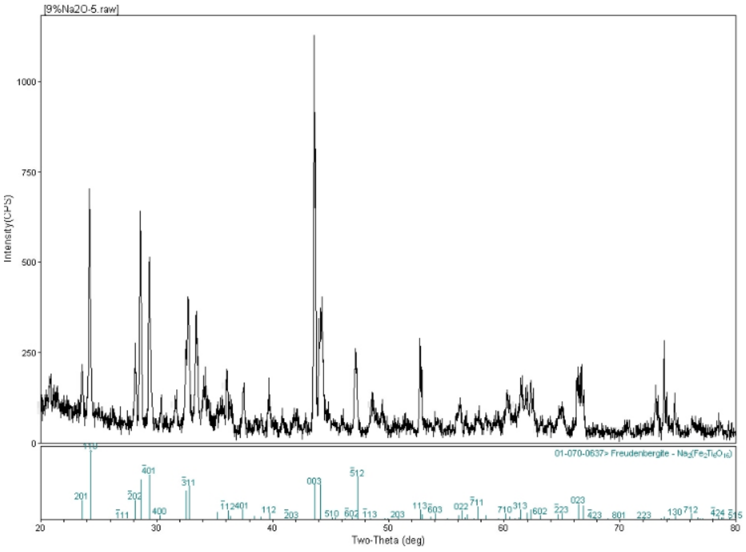

9Na2O – 60min

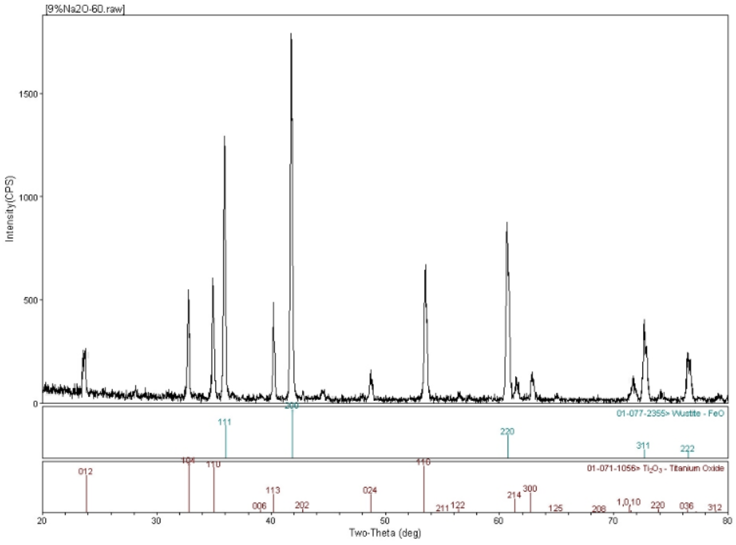

Supplementary 3. The refinement of each mineral phase in XRD pattern of slag samples shown in Fig 3e and 3f using JADE program.

Ilmenite (light grey)

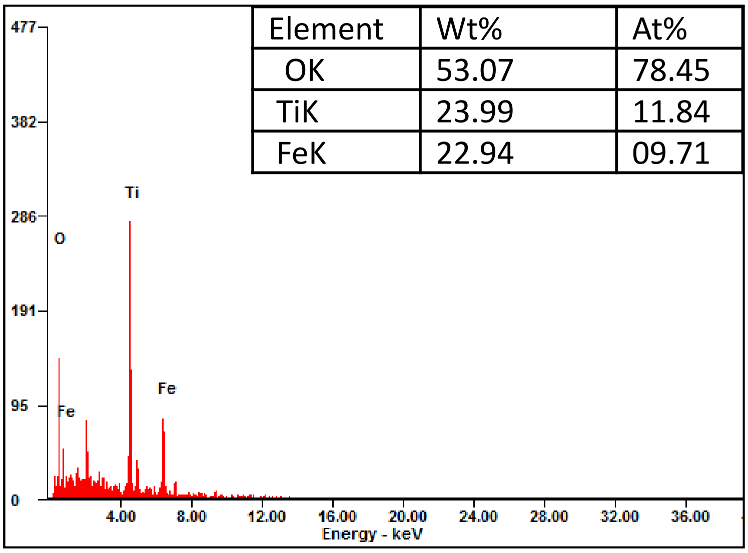

Element	Wt%	At%
OK	53.07	78.45
TiK	23.99	11.84
FeK	22.94	09.71

psb (grey)

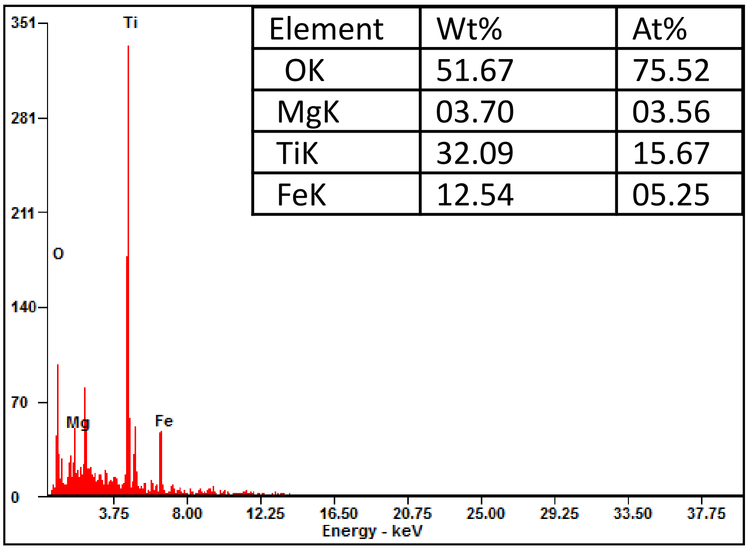

Element	Wt%	At%
OK	51.67	75.52
MgK	03.70	03.56
TiK	32.09	15.67
FeK	12.54	05.25

glass (dark)

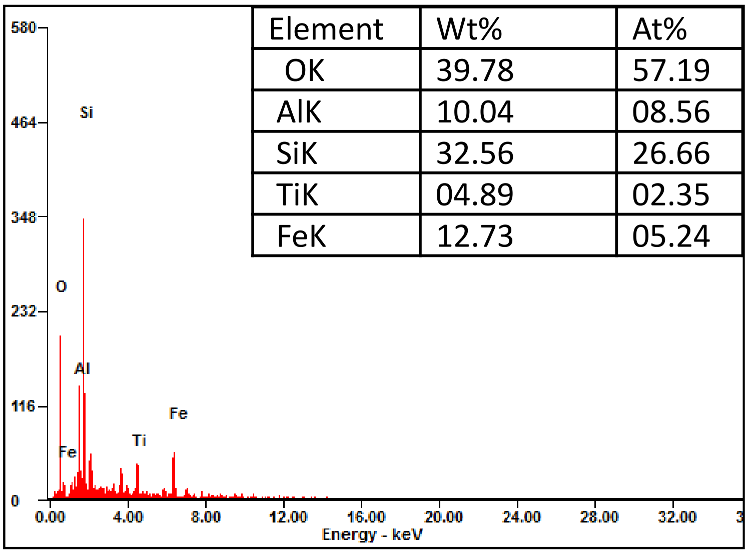

Element	Wt%	At%
OK	39.78	57.19
AlK	10.04	08.56
SiK	32.56	26.66
TiK	04.89	02.35
FeK	12.73	05.24

No flux

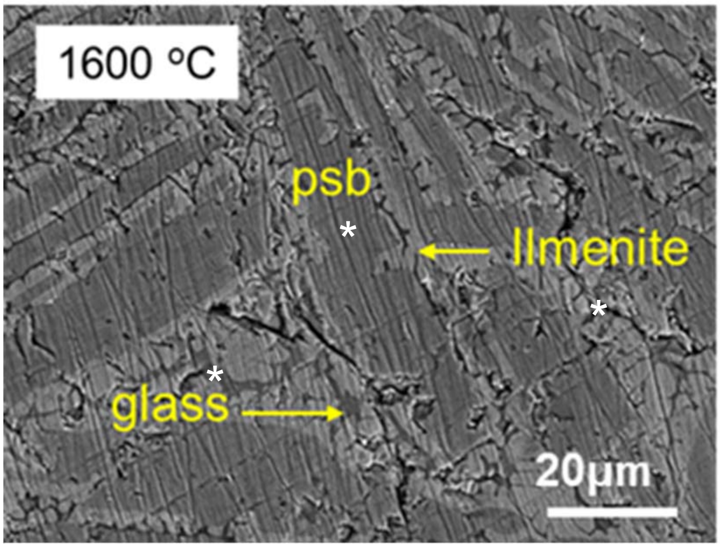

Supplementary 4. The EDS spectra of phases in slag samples shown in Figure 4.

ilmenite (light grey)

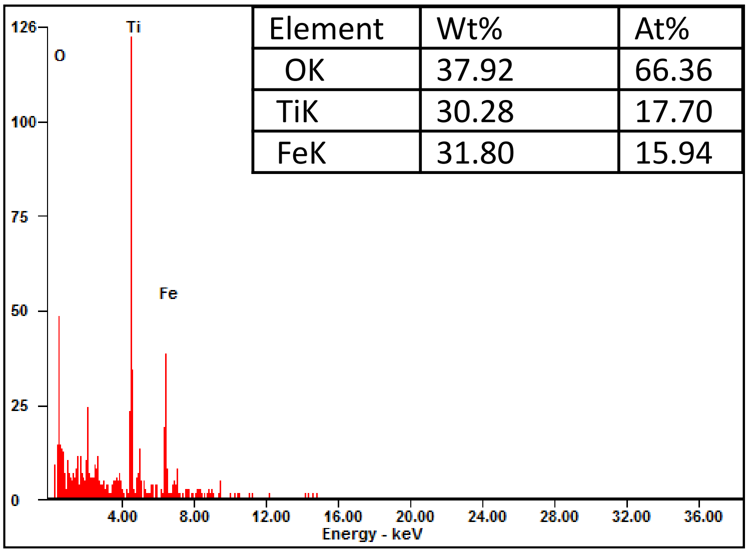

Element	Wt%	At%
OK	37.92	66.36
TiK	30.28	17.70
FeK	31.80	15.94

psb (grey)

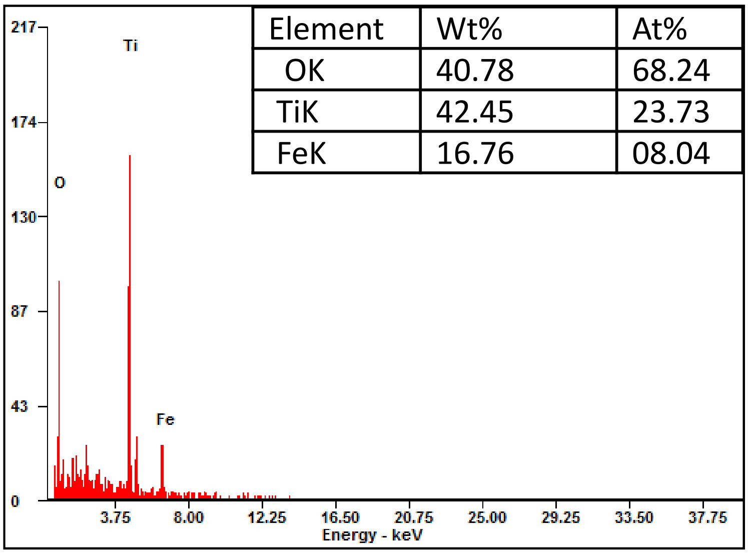

Element	Wt%	At%
OK	40.78	68.24
TiK	42.45	23.73
FeK	16.76	08.04

3% SiO_2

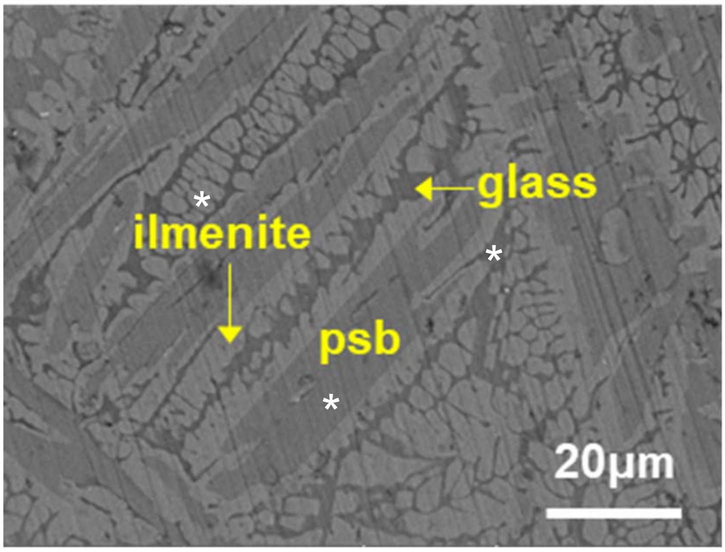

glass (dark)

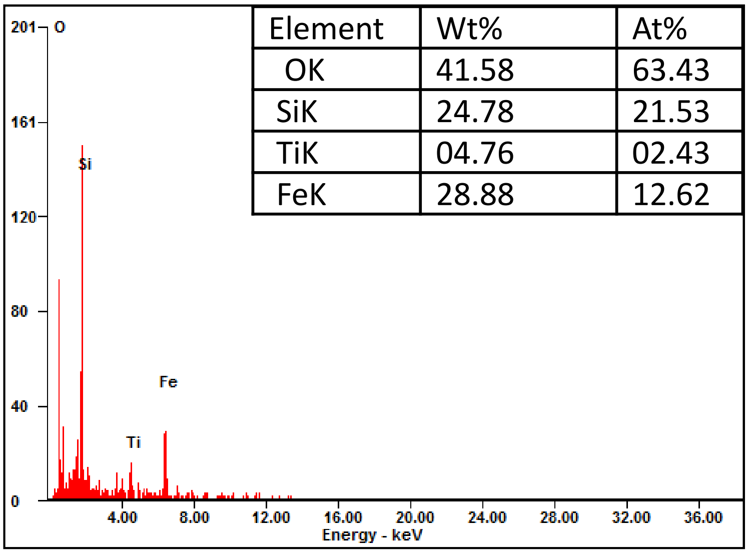

Element	Wt%	At%
OK	41.58	63.43
SiK	24.78	21.53
TiK	04.76	02.43
FeK	28.88	12.62

Supplementary 4. The EDS spectra of phases in slag samples shown in Figure 4.

ilmenite (light grey)

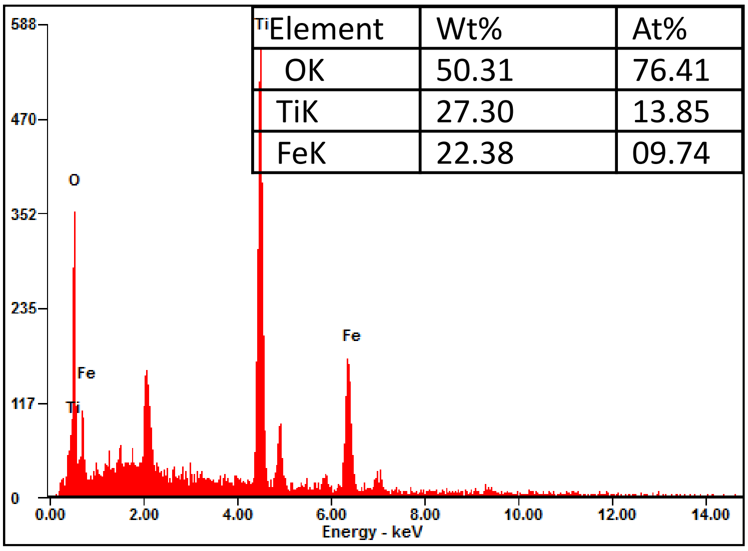

Element	Wt%	At%
OK	50.31	76.41
TiK	27.30	13.85
FeK	22.38	09.74

psb (grey)

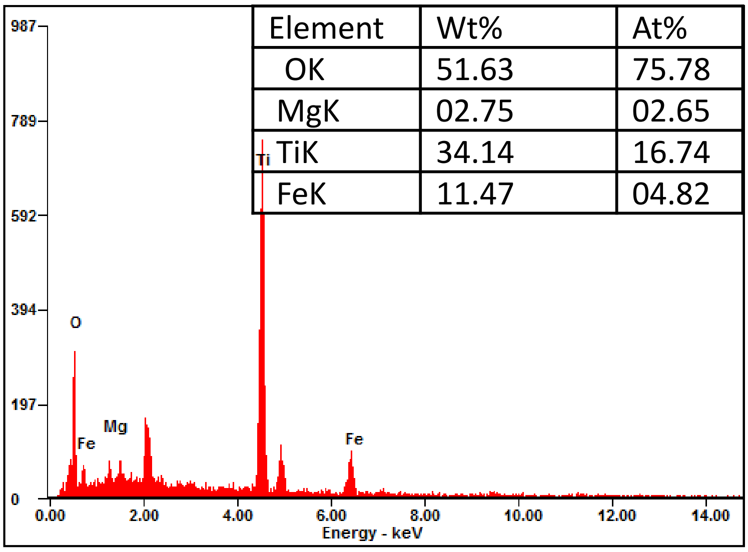

Element	Wt%	At%
OK	51.63	75.78
MgK	02.75	02.65
TiK	34.14	16.74
FeK	11.47	04.82

$6\% \ SiO_2$

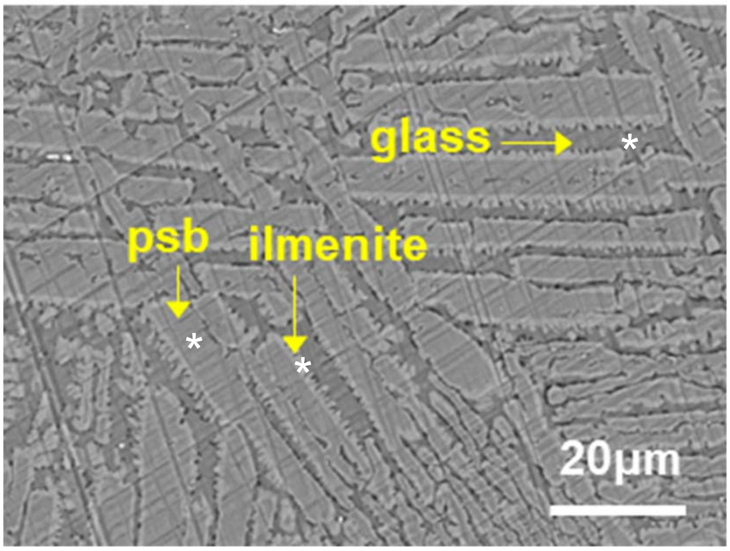

glass (dark)

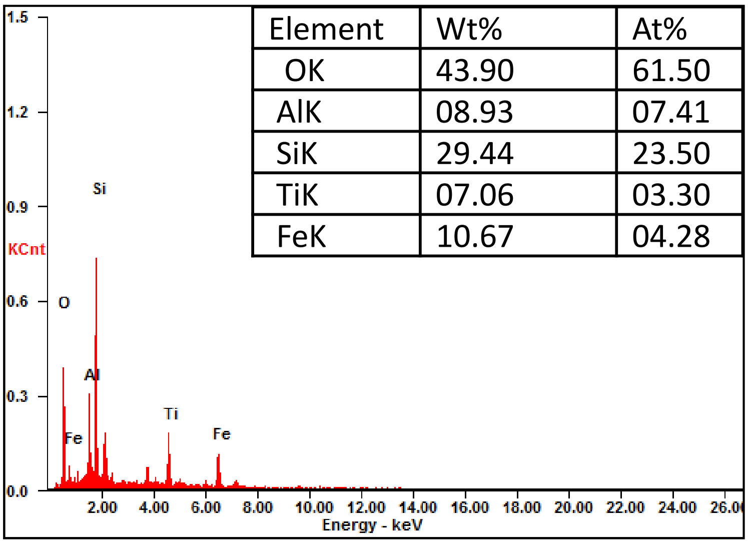

Element	Wt%	At%
OK	43.90	61.50
AlK	08.93	07.41
SiK	29.44	23.50
TiK	07.06	03.30
FeK	10.67	04.28

Supplementary 4. The EDS spectra of phases in slag samples shown in Figure 4.

ilmenite (light grey)

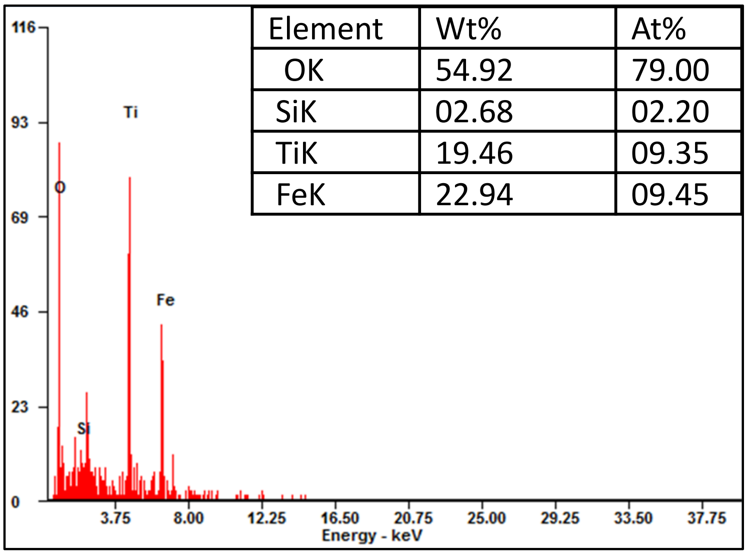

Element	Wt%	At%
OK	54.92	79.00
SiK	02.68	02.20
TiK	19.46	09.35
FeK	22.94	09.45

psb (grey)

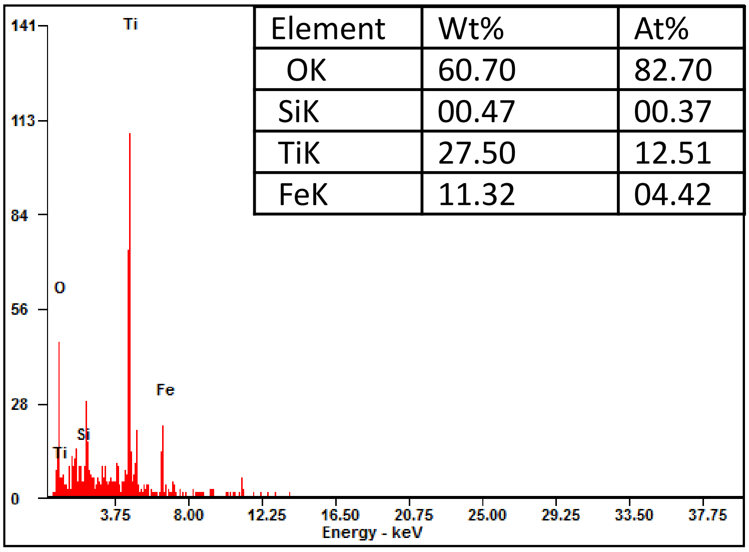

Element	Wt%	At%
OK	60.70	82.70
SiK	00.47	00.37
TiK	27.50	12.51
FeK	11.32	04.42

9% SiO_2

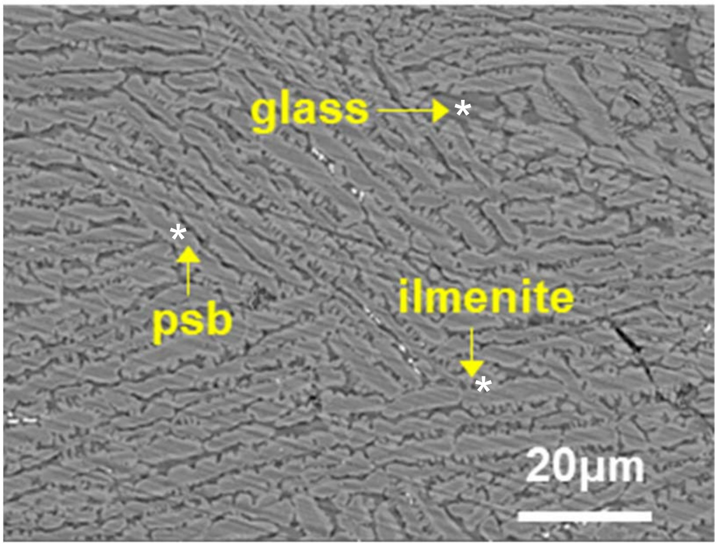

glass (dark)

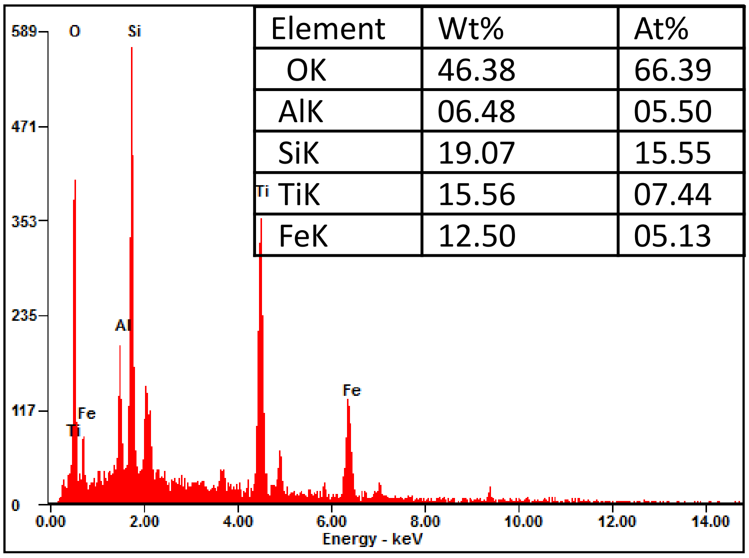

Element	Wt%	At%
OK	46.38	66.39
AlK	06.48	05.50
SiK	19.07	15.55
TiK	15.56	07.44
FeK	12.50	05.13

Supplementary 4. The EDS spectra of phases in slag samples shown in Figure 4.

ilmenite (light grey)

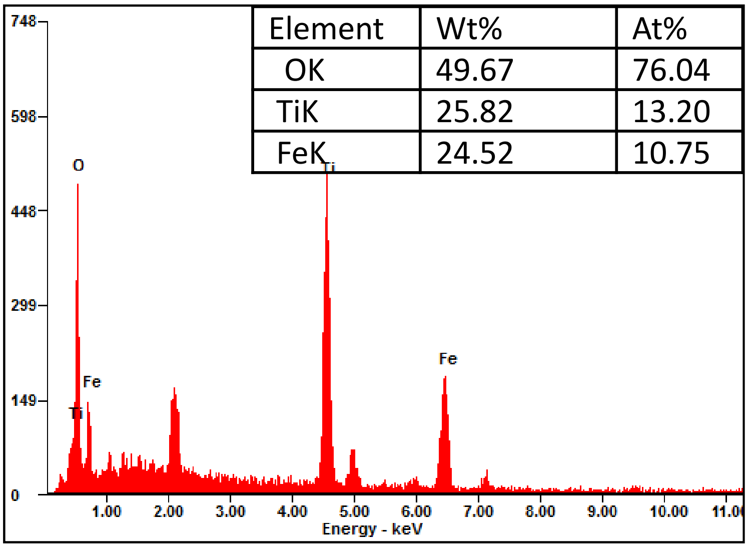

Element	Wt%	At%
OK	49.67	76.04
TiK	25.82	13.20
FeK	24.52	10.75

psb (grey)

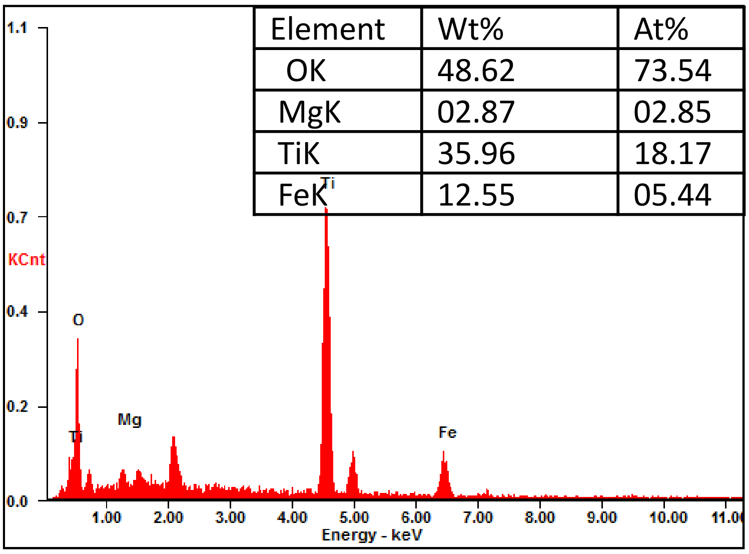

Element	Wt%	At%
OK	48.62	73.54
MgK	02.87	02.85
TiK	35.96	18.17
FeK	12.55	05.44

3% Na_2O

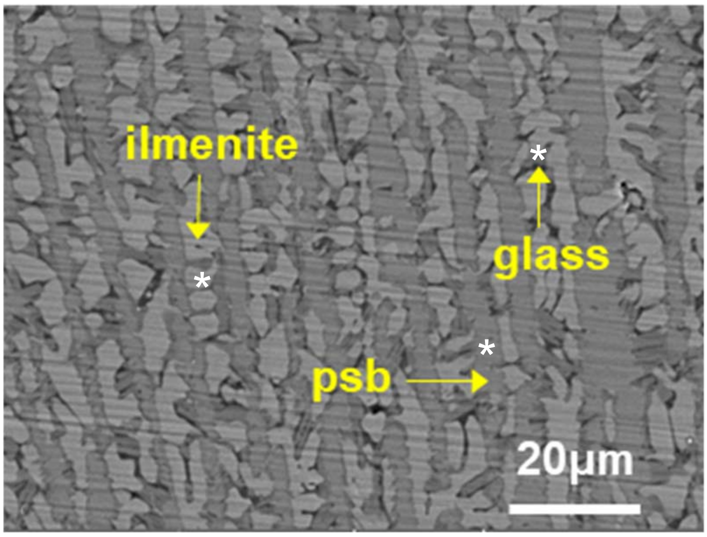

glass (dark)

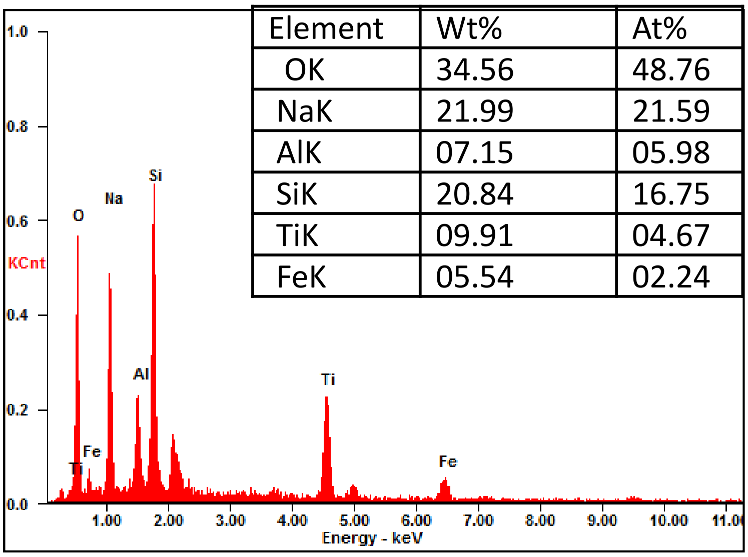

Element	Wt%	At%
OK	34.56	48.76
NaK	21.99	21.59
AlK	07.15	05.98
SiK	20.84	16.75
TiK	09.91	04.67
FeK	05.54	02.24

Supplementary 4. The EDS spectra of phases in slag samples shown in Figure 4.

ilmenite (light grey)

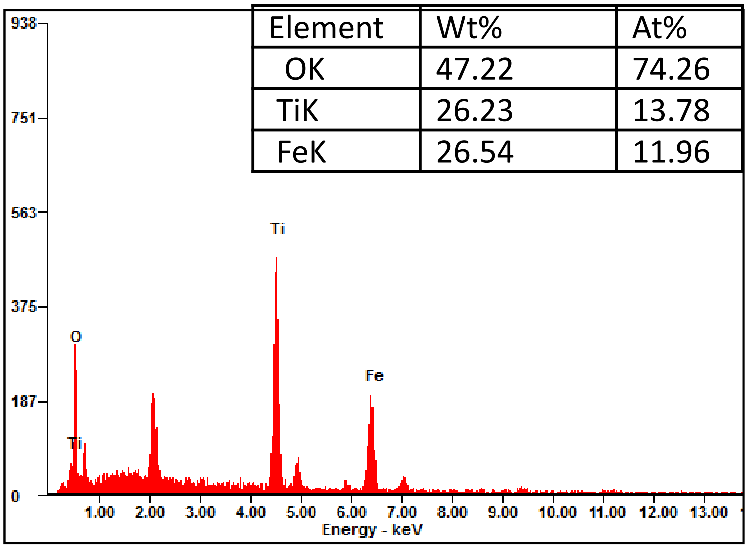

Element	Wt%	At%
OK	47.22	74.26
TiK	26.23	13.78
FeK	26.54	11.96

psb (grey)

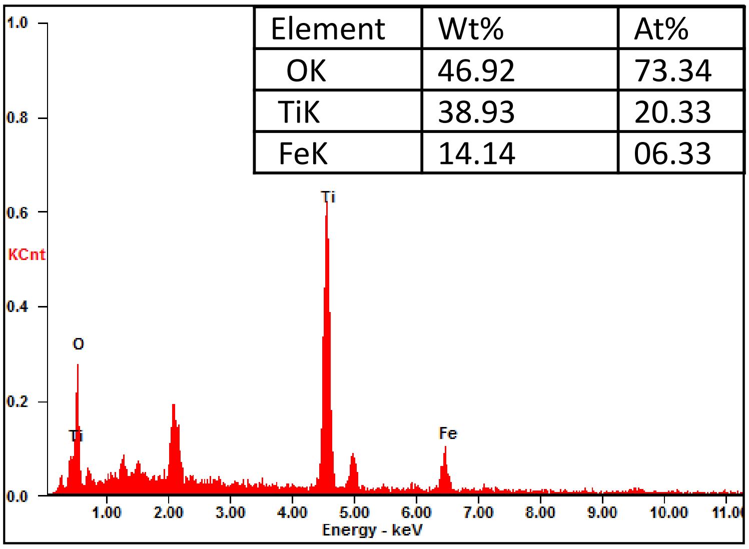

Element	Wt%	At%
OK	46.92	73.34
TiK	38.93	20.33
FeK	14.14	06.33

6% Na_2O

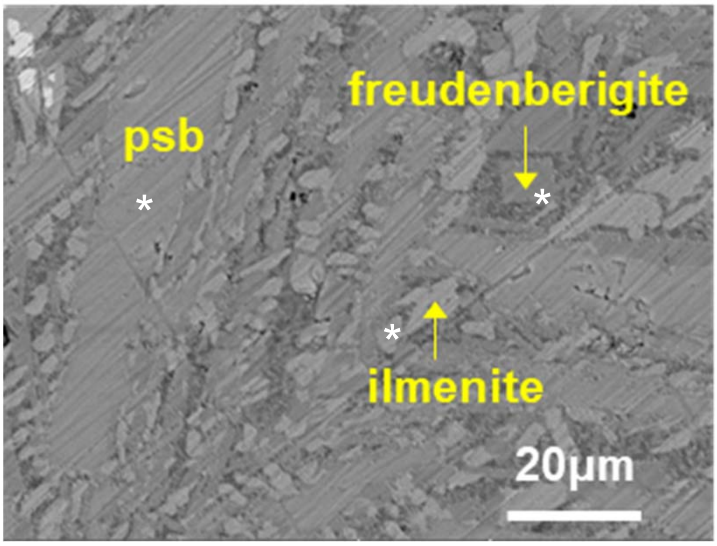

freudenberigite (dark grey)

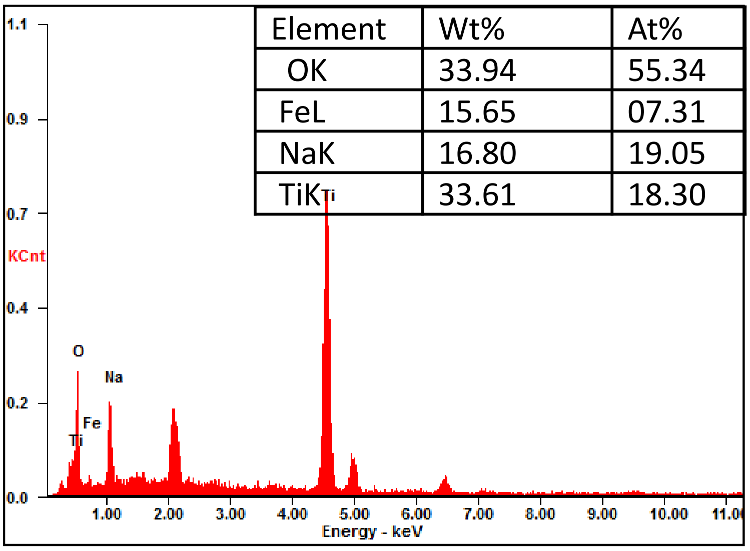

Element	Wt%	At%
OK	33.94	55.34
FeL	15.65	07.31
NaK	16.80	19.05
TiK	33.61	18.30

Supplementary 4. The EDS spectra of phases in slag samples shown in Figure 4.

freudenberigite

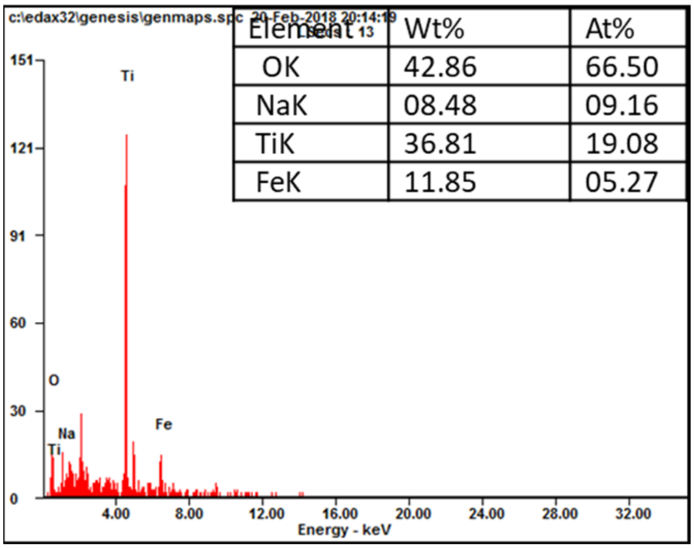

Element	Wt%	At%
OK	42.86	66.50
NaK	08.48	09.16
TiK	36.81	19.08
FeK	11.85	05.27

9% Na_2O

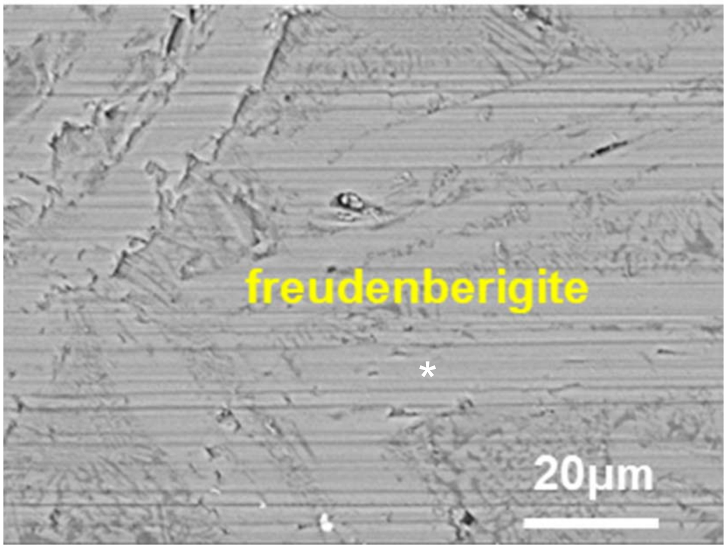

Supplementary 4. The EDS spectra of phases in slag samples shown in Figure 4.

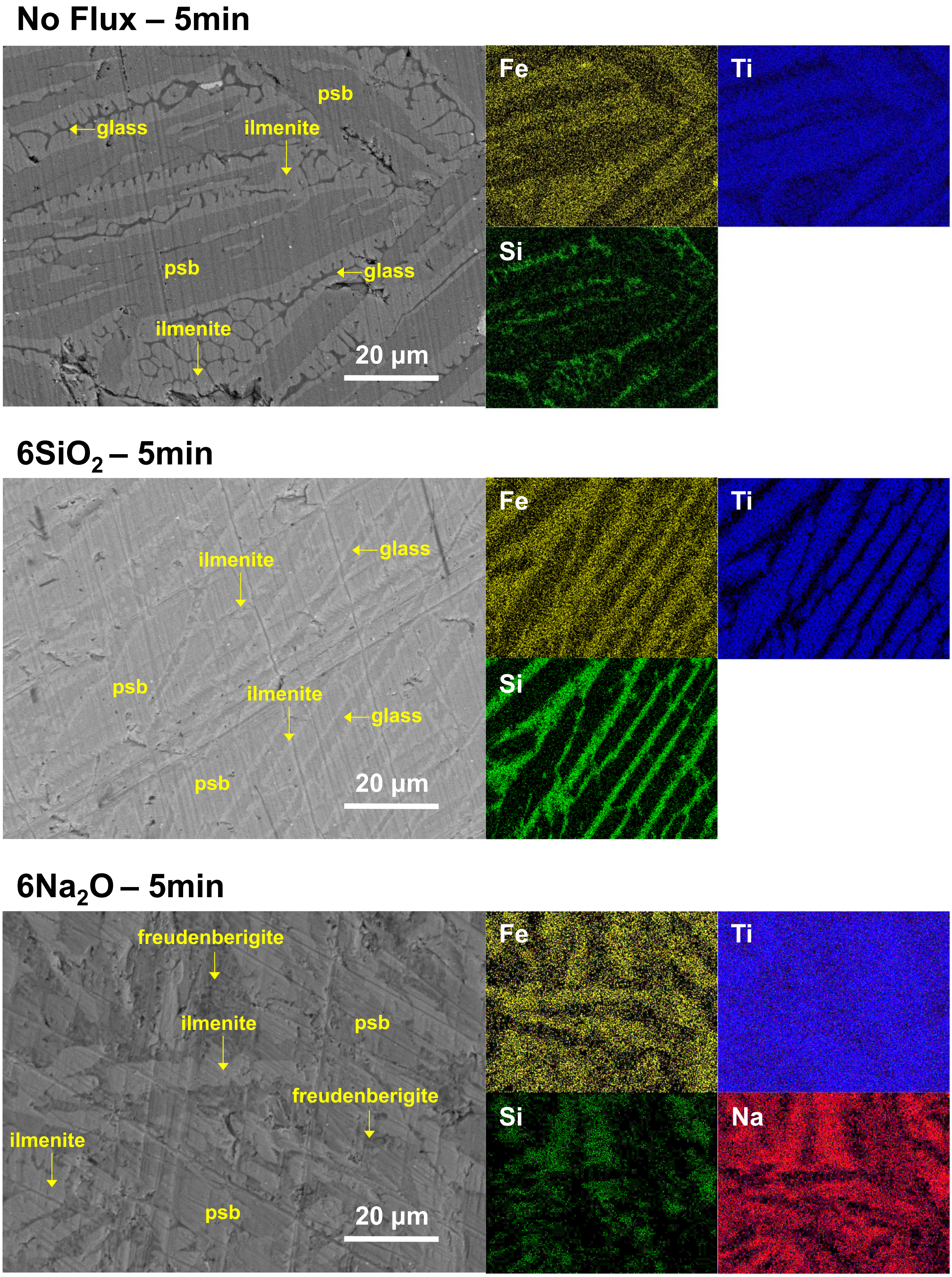

Supplementary 5. The element mapping analysis results for each mineral phase in slag samples.
